# Security Authentication Scheme for Vehicle-to-Everything Computing Task Offloading Environments

**DOI:** 10.3390/s25206428

**Published:** 2025-10-17

**Authors:** Yubao Liu, Chenhao Li, Quanchao Sun, Haiyue Jiang

**Affiliations:** College of Computer Science and Technology, Changchun University, Changchun 130012, China; 230702305@mails.ccu.edu.cn (C.L.); 241503556@mails.ccu.edu.cn (Q.S.); 241501500@mails.ccu.edu.cn (H.J.)

**Keywords:** computational task offloading, V2X, authentication, AVISPA, NS-3

## Abstract

Computational task offloading is a key technology in the field of vehicle-to-everything (V2X) communication, where security issues represent a core challenge throughout the offloading process. We must ensure the legitimacy of both the offloading entity (requesting vehicle) and the offloader (edge server or assisting vehicle), as well as the confidentiality and integrity of task data during transmission and processing. To this end, we propose a security authentication scheme for the V2X computational task offloading environment. We conducted rigorous formal and informal analyses of the scheme, supplemented by verification using the formal security verification tool AVISPA. This demonstrates that the proposed scheme possesses fundamental security properties in the V2X environment, capable of resisting various threats and attacks. Furthermore, compared to other related authentication schemes, our proposed solution exhibits favorable performance in terms of computational and communication overhead. Finally, we conducted network simulations using NS-3 to evaluate the scheme’s performance at the network layer. Overall, the proposed scheme provides reliable and scalable security guarantees tailored to the requirements of computing task offloading in V2X environments.

## 1. Introduction

Vehicle-to-everything (V2X) computing task offloading is an indispensable enabling technology for intelligent transportation systems, serving as the bridge connecting vehicle intelligence with smart traffic networks. In vehicle-to-everything (V2X) scenarios, computational tasks requiring offloading can be categorized into three types: computation-intensive, latency-sensitive, and hybrid. Computation-intensive tasks include map modeling and updating, environmental perception and fusion, and path planning. While these tasks do not demand high latency, they require substantial computational power ranging from hundreds of GOPS to several TOPS. Therefore, they should be offloaded to edge servers or cloud centers with richer computational resources for execution. Latency-sensitive tasks encompass collision warnings, collaborative perception sharing, and real-time vehicle platooning. While their computational demands are not exceptionally high, they impose extremely stringent latency requirements, typically demanding end-to-end latency between 10 milliseconds and 100 milliseconds, or even less than 10 milliseconds. Hybrid tasks encompass real-time decision-making and driver assistance (such as lane changes or ramp merging based on edge-server-enhanced visual perception). These tasks fall between the preceding two categories in terms of both computational power and latency requirements.

To address the challenges posed by traditional cloud computing—which cannot meet the stringent requirements of V2X for low latency, high channel bandwidth, and robust privacy and security—edge computing has emerged as a solution [1]. Edge computing addresses cloud computing’s limitations by relocating computational power and storage resources closer to the network edge—near the data source [1,2]. This enables rapid processing and timely responses to computational tasks while alleviating single-point pressure on cloud centers, establishing a complementary and collaborative relationship with the cloud [3]. Offloading V2X computing tasks onto edge computing infrastructure is a critical operation. By transferring vehicle-generated computational tasks to edge servers, it leverages their processing power and storage resources to handle task data and receive results [3,4]. This approach overcomes terminal computing limitations while meeting low-latency application demands. However, offloading significantly expands the attack surface. First, attackers can impersonate legitimate edge servers. Requesting vehicles may unload computational tasks—containing sensitive identity information, location data, sensor readings, or even control commands—onto these disguised servers. This enables attackers to steal private data or return erroneous results, potentially causing requesting vehicles to make fatal misjudgments. Second, attackers may impersonate legitimate requesting vehicles to consume edge server computational resources. As the number of spoofed requesting vehicle nodes increases, server resources can be rapidly depleted, denying service to legitimate requesting vehicles. Furthermore, the task offloading process always occurs over open channels, exposing transmitted messages to eavesdropping, interception, forgery, and tampering by attackers—potentially causing severe consequences [5]. Therefore, authentication between relevant entities is an absolutely essential first line of defense before implementing computational task offloading. To this end, we need to design an authentication key exchange scheme to ensure the legitimacy of entities participating in computational task offloading and the confidentiality of the offloading process.

In this paper, the computational task offloading scenarios we investigate include offloading between the requesting vehicle and an edge server (V2I), as well as offloading between the requesting vehicle and an auxiliary vehicle (V2V). The auxiliary vehicle can leverage its idle resources to serve the requesting vehicle, thereby extending the coverage range of the edge to some extent. In the V2I computational offloading scenario, roadside units (RSUs) serve as information aggregation and distribution hubs, responsible for relaying messages between vehicles within their jurisdiction and the edge server.

The primary objective of this paper is to design an authentication scheme for the vehicle-to-everything (V2X) computing task offloading environment. This scheme ensures the security of communication entities involved in task offloading and the offloading process itself, while minimizing computational overhead to guarantee high efficiency. Our proposed secure authentication scheme features the following characteristics:

- Includes four-party authentication involving the requesting vehicles, roadside units (RSUs), edge servers (ESs), and the cloud management center (CMC—all entities accessing the vehicle-to-everything network must first register here), as well as three-party authentication between the requesting vehicles, auxiliary vehicles, and the cloud management center.
- This solution employs lightweight cryptographic techniques and operations such as elliptic curve cryptography, hash functions, physically unclonable function (PUF), and XOR. This approach provides security protection while minimizing computational and communication overhead associated with task offloading.
- The proposed scheme underwent formal analysis using BAN logic and the ROR model and was validated through the AVISPA tool. It has been demonstrated to possess reliable security properties and can withstand potential attacks on vehicle-to-everything (V2X) networks.
- We compared our proposed scheme with existing schemes in the field of vehicle-to-everything (V2X) communication. Our scheme demonstrates favorable performance in terms of authentication overhead.

## 2. Related Work

The security framework in the vehicle-to-everything (V2X) environment is built upon two core issues: identity authentication and behavioral trustworthiness. Identity authentication serves as the foundation for ensuring the legitimacy of communicating entities, while behavioral trustworthiness is evaluated through trust management models, aiming to determine the reliability of authenticated entities’ actions. To defend against unpredictable, potentially malicious attacks in the heterogeneous V2X environment and enhance the “trusted decision-making” capabilities at the application layer, numerous scholars have proposed relevant solutions.

Regarding identity authentication, Li et al. [5] designed an authentication protocol for V2X with lightweight characteristics. The protocol employs low-overhead operations like HASH and XOR operations, reducing computational overhead and enhancing authentication efficiency. However, it does not support forward secrecy and fails to account for the impact of real-world physical layer attacks on On-Board Units (OBUs). Gupta et al. [6] proposed a hierarchical authentication mechanism based on identity-based cryptography or short signature algorithms, integrating network slicing technology to provide differentiated bandwidth for various service data. By processing authentication requests at MEC nodes, it reduces backhaul time but may introduce single-point-of-failure risks. Han et al. [7] proposed an anonymous authentication scheme based on fog computing. However, the protocol employs computationally complex bilinear pairing, which is unsuitable for resource-constrained V2X environments and low-latency requirements. Yang et al. [8] introduced an Edge-Assisted Distributed Authentication (EADA) architecture, which also offloads authentication capabilities to RSUs and base stations while tailoring vehicle authentication based on vehicle status. Cui et al. [9] proposed a scalable in-vehicle network authentication scheme for multi-cloud environments, employing a Cloud Broker (CB) managed by the TA to connect all cloud services, thereby shielding vehicle users from the complexity of selecting cloud services. Bagga et al. [10] designed a robust authentication and key establishment scheme for intelligent transportation systems (ITSs) that allows new vehicles to dynamically join RSUs after initial deployment, while reducing the management burden on the TA. Vinoth et al. [11] designed a secure key negotiation scheme for the Industrial Internet of Things (IoT) between a single user and multiple sensor devices. Adopting the key sharing concept and leveraging the Chinese Remainder Theorem (CRT) from number theory, the scheme constructs group keys for multiple sensor devices while being highly suitable for resource-constrained environments. Zhou et al. [12] identified computational inconsistencies in the security proofs of Ali’s [13] CLCPPA protocol for V2I communications. They improved upon Ali et al.’s work and demonstrated that the enhanced CLCPPA effectively defends against signature forgery attacks. Cao et al. [14] proposed a lattice-based group signature authentication protocol, implementing forward secrecy using a Bonsai-tree signature scheme. Prateek et al. introduced a privacy-preserving authentication scheme based on quantum key distribution protocols for V2I communications. Son et al. [15] proposed a blockchain-based VANET handover authentication protocol where vehicles perform only lightweight computations during zone transitions, demonstrating the protocol’s feasibility through formal tools and simulation experiments. Yang et al. [16] proposed an ECC-based anonymous authentication scheme with a two-stage process: initial authentication followed by subsequent authentication phases. Subsequent authentication relies on the initial authentication, but this places significant authentication pressure on RSUs, especially when authenticating numerous vehicles, potentially causing performance bottlenecks. Wei et al. [17] addressed the single point of failure issue in root-TA models by adopting a multi-TA architecture. They implemented an identity authentication list within sub-TAs and employed the Cuckoo Filter to optimize retrieval processes. Novelly, they utilized Lagrange’s Interpolation Theorem during mutual authentication to establish session keys. Similarly, the multi-TA model is referenced in [18]. Awais et al. [19] propose a lightweight key exchange scheme using PUFs for n vehicles in VANETs, resisting physical attack risks. It employs hash encryption and XOR operations to reduce computational overhead and communication costs. This scheme is proven secure under the ROR model and satisfies relevant security properties. Mulambia et al. [20] introduced a blockchain-based VANET architecture comprising three layers: the message propagation layer, the management layer, and the blockchain layer. Roadside units (RSUs) function as authentication managers, verifying vehicle information. Management-layer RSUs broadcast their known authentication data to other RSUs at the same layer. All RSUs submit hashed records to the blockchain, thereby reducing the propagation of malicious information by rogue nodes. However, this approach incurs increasing latency as the number of transactions within blocks grows, necessitating a trade-off with security. Yu et al. [21] proposed RLBA-UAV, a blockchain-based authentication and key establishment scheme addressing privacy and security challenges in sensor and IoT data transmission for UAVs. Leveraging UAVs’ PUF capabilities, the scheme demonstrated proven security properties via ROR and AVISPA models while exhibiting efficiency compared to existing approaches. These studies lay a solid foundation for our exploration in the IOV domain and hold significant importance. Othman et al. [22] proposed a secure authentication protocol based on Physical Unclonable Functions (PUFs). Vehicles and road side units (RSUs) can use embedded PUFs to construct polynomial shared keys. The use of PUFs prevents attackers from impersonating legitimate entities. Additionally, the authors propose a revocation mechanism “SGKD”, supporting both temporary and permanent revocation, reducing the time complexity from O(${log}^{N}$) to O(1). For vehicle-to-vehicle communication, the protocol distinguishes between unicast fast authentication and broadcast secure message transmission scenarios. It also demonstrates strong resistance against desynchronization attacks. Wu et al. [23] proposed a lightweight vehicle social network security authentication protocol based on fog nodes. Unfortunately, Li et al. [24] discovered that their authentication protocol contained threats, failing to resist insider attacks and smart card theft attacks, and lacked perfect forward secrecy. Li et al. improved Wu’s scheme in [24] and proved the effectiveness of their enhanced scheme.

Regarding behavioral trustworthiness, Liu et al. [25] proposed a Trust Cascade-based emergency message dissemination (TCEMD) model in [25], which comprehensively considers the synergistic effects of multiple factors. This model efficiently integrates entity-oriented trust values into data-oriented trust assessment and consolidates the analysis of messages reporting different states of emergency events. It addresses the shortcomings of existing models and demonstrates its superiority through a series of simulations and analyses. In 2021, Liu et al. [26] proposed an innovative Privacy-Preserving Trust Management (PPTM) scheme for emergency message dissemination in Space–Air–Ground Integrated Networks. The paper thoroughly demonstrates the correctness, precise management, and robust practicality advantages of the PPTM scheme. Through simulation analysis, it proves that the scheme achieves precise trust management and strong conditional privacy protection while maintaining low communication overhead. Recently, Liu et al. proposed a novel Privacy-Preserving Reputation Updating (PPRU) scheme in [27] to address the pressure load issue on the TA side in cloud-assisted vehicle-to-everything (V2X) networks. This scheme leverages ECC and the Paillier algorithm, delegating reputation feedback collection and preprocessing to the cloud service provider (CSP). This significantly reduces the computational burden on the TA while supporting weighted average calculations of encrypted ratings, enabling more robust reputation management. Simulation analysis demonstrates that the scheme achieves strong privacy protection against potential attacks with acceptable computational and communication overhead.

In this paper, we aim to propose an authentication scheme tailored to the vehicle-to-everything (V2X) computing task offloading environment, laying a foundational trust base for subsequent behavioral trustworthiness issues within such environments. We have summarized some of the proposed schemes in Table 1.

## 3. Preliminaries

### 3.1. Elliptic Curve Cryptography

We select a non-singular elliptic curve E, whose form is $y^{2}=x^{3}+ax+b(mod\;q)$. It is defined over the finite field $F_{q}$, where q denotes a large prime number, $a,\;b\in F_{q}=\;\{0,\;1,\;2,\;\ldots ,\;q-1\}$ and satisfies $4a^{3}+27b^{2}\neq 0$. The points on the curve together with the point at infinity O form an Abelian group. Given a base point P, we have P + O = P. Elliptic curve cryptography relies on two major computational challenges. The first is ECDLP: given a base point P and a point Q=d×P on an elliptic curve, where $d\in Z_{p}^{*}$, p is the order of the cyclic subgroup generated by the point P, that is, the smallest positive integer *p* such that p×P=O. It is computationally difficult to find d. The second is ECDHP: given a base point P, a point A, and a point B, $A=d_{A}\times P,B=d_{B}\times P$, where $d_{A}$, $d_{B}\in Z_{p}^{*}$, it is computationally difficult to find the common key $C=d_{A}\times d_{B}\times P$.

### 3.2. Physically Unclonable Function (PUF)

PUF is a security technology based on hardware physical characteristics that generates a unique and non-replicable “fingerprint” for each chip or physical device [31]. PUF primarily leverages minute manufacturing variations inherent in hardware production to ensure each device produces a unique PUF response. The unpredictability of the manufacturing process also renders it non-cloneable [32]. PUF is currently widely discussed in the field of protecting IoT privacy and security. For telematics computing task offloading, embedding PUF within the integrated circuit of a vehicle’s On-Board Unit (OBU) device can resist physical attacks. Its security capability is based on dynamic challenge–response interactions, generating distinct responses to different challenges. Compared to traditional chips, PUFs consume minimal hardware resources, with negligible area overhead and dynamic power consumption [32]. This makes them exceptionally well-suited for the V2X environment, effectively addressing the resource constraints and power sensitivity challenges inherent in V2X devices. We adopted the “SRAM PUF” in our proposed scheme because of its high maturity. It can utilize a Fuzzy Extractor to address bit flipping issues caused by temperature and voltage variations, ensuring stable output. Moreover, its extremely fast response speed makes it well-suited for the real-time requirements of communication authentication.

### 3.3. Hash Function

Hash functions are fundamental tools in cryptography. They accept input messages of arbitrary length and produce a fixed-length “fingerprint” output, always generating the same output for identical inputs. Hash functions exhibit strong resistance to preimage attacks and collision attacks [33]. Their unique “avalanche effect” and computational efficiency also make them widely used cryptographic techniques in cryptography (such as digital signatures, blockchain, and HMAC).

### 3.4. Adversary Threat Model

We consider employing two classical threat models—the DY model proposed by Dolev and Yao [34] and the CK model proposed by Canetti and Krawczyk [35]—to define the capabilities of adversary A in our proposed scheme. The CK model treats messages transmitted between protocol entities as abstract symbols, focusing on the security of protocol logic while assuming the attacker has complete control over the network. The CK model extends the attacker’s capabilities beyond the DY model by allowing selective sabotage of specific sessions and access to session-specific temporary keys. This makes the attack scenario more realistic, encompassing practical risks such as network attacks, key compromises, and insider threats. Therefore, we define the capabilities possessed by adversary A in our paper as follows:

- A can eavesdrop on, delete, tamper with, replay, or forge messages transmitted over the public channel.
- A can steal a vehicle’s OBU and extract stored information through physical attacks.
- A can impersonate legitimate users.
- A can guess temporary secret values through enumeration attacks.
- A can exploit key leaks and session vulnerabilities to launch attacks.

### 3.5. V2X Computing Task Offloading System Model

**Figure 1:**
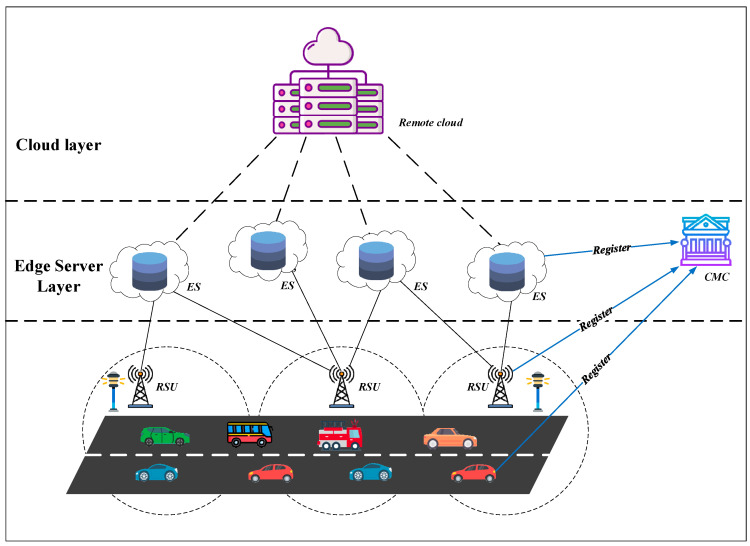
Vehicle-to-everything computing task offloading system model.

The vehicle-to-everything (V2X) computing task offloading system model we established is shown in Figure 1. The entities involved in offloading include vehicles, roadside units (RSUs), edge servers (ESs), and the cloud management center (CMC).

- For each vehicle entity, the On-Board Unit (OBU) serves as its core device, providing the hardware foundation for the vehicle to communicate with the external environment while possessing a certain degree of data processing and decision-making capability.
- Roadside units (RSUs) function as relay nodes and collaborative nodes within the vehicle-to-everything (V2X) computing task offloading system. They collect offloading requests issued by vehicles within the managed area and transmit status information of connected edge servers and other auxiliary vehicles in the region to the requesting vehicle. Additionally, they distribute computing task data processed by edge servers and related instructions to the managed area for centralized coordination.
- Edge servers (ESs) possess greater storage capacity and computational power than vehicles and RSUs. They handle computational tasks offloaded by vehicles, addressing the constraints of limited vehicle resources while enabling rapid response.
- The cloud management center (CMC) serves as the cloud service provider and management authority for the entire vehicle-to-everything (V2X) computing task offloading system. All entities must register here before entering the system to obtain their unique, legitimate credentials.

## 4. Proposed Scheme

### 4.1. Initialization Phase

Step 1: CMC selects a large prime number q and defines an elliptic curve E over the finite field Fq: $y^{2}=x^{3}+ax+b\;mod\;q(where\;4a^{3}+27b^{2}\neq 0)$. Then, a point P is chosen as the base point.

Step 2: CMC randomly selects a number $S\in Z_{p}^{*}$ as the system private key and calculates the system public key ${PK}_{sys}=S\times P$.

Step 3: CMC selects a set of secure hash functions: $H_{1}:{\left\{0,1\right\}}^{*}\to {\left\{0,1\right\}}^{length}$, $H_{2}:{\left\{0,\;1\right\}}^{*}\to Z_{p}^{*}$, $H_{3}:E(Fq)\to {\left\{0,1\right\}}^{*}$, $H_{4}:(E\left(Fq\right),\;{\left\{0,1\right\}}^{*})\to {\left\{0,1\right\}}^{length}$.

Finally, CMC publishes the parameters $\left\{a,b,q,P,H(),{PK}_{sys}\right\}$ to the entire system.

### 4.2. Entity Registration Phase

The symbols used in the scheme are described in Table 2.

#### 4.2.1. Vehicle Registration Phase (VRP)

VRP-1: The vehicle issues a registration request, and the CMC generates a set of PUF challenges $\left\{{Cha}_{1},{Cha}_{2},{Cha}_{3}\ldots \right\}$ and transmits them to the requesting vehicle via a secure channel.

VRP-2: Upon receiving the challenge set, the vehicle uses the PUF to compute the response set $PUF\left({Cha}_{h}\right)={Res}_{h}(h=1,2,3\ldots )$ and stores this challenge–response pair. The user selects and inputs their name ${ID}_{U}$ and password ${PWD}_{U}$, then enters their fingerprint information ${fp}_{U}$ into the vehicle. The vehicle computes $Gen({fp}_{U})=(\sigma ,\tau )$, then selects a challenge–response pair $\left\{{Cha}_{i},{Res}_{i}\right\}$ and further calculates ${PID}_{U}^{i}=H_{1}({ID}_{U}\|\sigma \|{Res}_{i})$ and ${PPW}_{U}^{i}=H_{1}({PWD}_{U}\|{Res}_{i})$. Finally, it transmits ${VRM}_{1}=\{{ID}_{V}^{i},{PID}_{U}^{i},{PPW}_{U}^{i},{Cha}_{i},{Res}_{i}\}$ to the CMC.

VRP-3: Upon receiving ${VRM}_{1}$, CMC computes $X_{i}=H_{1}({ID}_{V}^{i}\|{PID}_{U}^{i}\|S\|{Res}_{i}),\;A_{i}=X_{i}⊕H_{1}({PID}_{U}^{i}\|{PPW}_{U}^{i}\|{ID}_{V}^{i}),\;B_{i}=H_{1}(X_{i}\|{PID}_{U}^{i}\|{PPW}_{U}^{i})$. It then selects a random number ${nonce}_{V}^{i}$ and calculates $S_{V}^{i}=\left(S\times H_{2}\left({ID}_{V}^{i}\|{PID}_{U}^{i}{\|nonce}_{V}^{i}\right)\right)mod\;q,\;{TID}_{V}^{i}=({ID}_{V}^{i}\|{PID}_{U}^{i})⊕H_{1}(S\|{ID}_{CMC})$. Finally, it transmits ${VRM}_{2}=\{A_{i},B_{i},S_{V}^{i},{TID}_{V}^{i}\}$ to the vehicle via the secure channel and stores the vehicle’s challenge response for {${Cha}_{i},{Res}_{i}$}.

VRP-4: Upon receiving ${VRM}_{2}$, the vehicle calculates $C_{i}=S_{V}^{i}⊕H_{2}({PID}_{U}^{i}\|{PPW}_{U}^{i}\|{Res}_{i})$ and stores $\left\{A_{i},B_{i},C_{i},{TID}_{V}^{i}\right\}$.

#### 4.2.2. RSU Registration Phase (RRP)

RRP-1: RSU sends ${RRM}_{1}=\{{ID}_{RSU}^{j}\}$ to CMC via the secure channel.

RRP-2: Upon receiving ${RRM}_{1}$, CMC computes $Y_{j}=H_{1}({ID}_{RSU}^{j}\|S)$, then selects a random number ${nonce}_{R}^{j}$, and further calculates $S_{R}^{j}=(S\cdot H_{2}({ID}_{RSU}^{j}\|{nonce}_{R}^{j}))mod\;q$ and ${TID}_{R}^{j}={ID}_{RSU}^{j}⊕H_{1}(S\left\|{ID}_{CMC}\right),{PK}_{RSU}^{j}=S_{R}^{j}\times P$. Finally, it transmits ${RRM}_{2}=\{Y_{j},S_{R}^{j},{TID}_{R}^{j}\}$ to the RSU via a secure channel and publishes ${PK}_{RSU}^{j}$ to the system.

RRP-3: After receiving the message ${RRM}_{2}$, the RSU computes $D_{j}=Y_{j}⊕{ID}_{RSU}^{j}$ and $E_{j}=S_{R}^{j}⊕H_{2}(Y_{j}\|{TID}_{R}^{j})$ and stores $\left\{D_{j},E_{j},{TID}_{R}^{j}\right\}$.

#### 4.2.3. ES Registration Phase (ERP)

RRP-1: ES sends ${ERM}_{1}=\{{ID}_{ES}^{k}\}$ to CMC via the secure channel.

RRP-2: Upon receiving ${RRM}_{1}$, CMC computes $Z_{k}=H_{1}({ID}_{ES}^{k}\|S)$, then selects a random number ${nonce}_{E}^{k}$, and further calculates $S_{E}^{k}=(S\cdot H_{2}({ID}_{ES}^{k}\|{nonce}_{E}^{k}))mod\;q$ and ${TID}_{E}^{k}={ID}_{ES}^{k}⊕H_{1}(S\left\|{ID}_{CMC}\right),\;{PK}_{ES}^{k}=S_{E}^{k}\times P$. Finally, it transmits ${RRM}_{2}=\{Z_{k},S_{E}^{k},{TID}_{E}^{k}\}$ to the RSU via a secure channel and publishes ${PK}_{ES}^{k}$ to the system.

RRP-3: After receiving the message ${RRM}_{2}$, the RSU computes $F_{k}=Z_{k}⊕{ID}_{ES}^{k}$ and $G_{k}=S_{E}^{k}⊕H_{2}(Z_{k}\|{TID}_{E}^{k})$ and stores $\left\{F_{k},G_{k},{TID}_{E}^{k}\right\}$.

### 4.3. Vehicle Login Phase (VLP)

When a vehicle enters the coverage area of its assigned RSU, it must log in to the RSU.

VLP-1: The user inputs their ${{ID}_{U}}^{*}$ and password ${{PWD}_{U}}^{*}$ and registers their fingerprint information ${{fp}_{U}}^{*}$. The vehicle sequentially calculates $Rep({{fp}_{U}}^{*},\tau )=\sigma^{*},\;{{PID}_{U}^{i}}^{*}=H_{1}({{ID}_{U}}^{*}\|\sigma^{*}\|{Res}_{i}),\;{{PPW}_{U}^{i}}^{*}=H_{1}({{PWD}_{U}}^{*}\left\|{Res}_{i}\right),\;{X_{i}}^{*}=A_{i}⊕H_{1}\left({{PID}_{U}^{i}}^{*}\left\|{{PPW}_{U}^{i}}^{*}\right\|{ID}_{V}^{i}\right),\;{B_{i}}^{*}=H_{1}({X_{i}}^{*}\|{{PID}_{U}^{i}}^{*}\|{{PPW}_{U}^{i}}^{*})$, verifies whether ${B_{i}}^{*}$ equals $B_{i}$; if equal, login successful; otherwise, immediately abort the login.

VLP-2: When a vehicle needs to offload computational tasks, it generates a request message Request and selects a random number α, where $\alpha \in Z_{p}^{*}$, then computes $\;M_{1}^{i}=\;\alpha \times P,\;M_{2}^{i}=\alpha \times {PK}_{RSU}^{j},\;D_{1}=(Request\left\|{TID}_{V}^{i}\right)⊕H_{3}\left(M_{2}^{i}\right);\;$next, it generates a timestamp $T_{1}$, continues calculating $Q=\alpha +H_{2}(H_{3}\left(M_{2}^{i}\right)\left\|T_{1}\right),\;D_{2}=H_{1}(Request\left\|T_{1}\right),$ and finally sends ${VLP}_{1}=$ {$M_{1}^{i},\;M_{2}^{i},\;D_{1},\;Q,\;D_{2}$} to the regional RSU.

VLP-3: Upon receiving ${VLP}_{1}$, the RSU first verifies the timestamp’s freshness, then calculates $M_{2}^{i}=S_{R}^{j}\times M_{1}^{i},({Request}^{*}\left\|{TID}_{V}^{i}\right)=D_{1}⊕H_{3}\left(M_{2}^{i}\right),D_{3}=H_{2}(H_{3}\left(M_{2}^{i}\right)\left\|T_{1}\right),{D_{2}}^{*}=H_{1}({Request}^{*}\left\|T_{1}\right)$, verifies whether Q×P equals $\left(M_{1}^{i}+D_{3}\times {PK}_{V}^{i}\right)$ and ${D_{2}}^{*}$ equals $D_{2}$; if they are equal, then RSU generates a response message Ack and a timestamp $T_{2}$, then calculates $D_{4}=(Ack\left\|T_{2}\right)⊕H_{3}\left(M_{2}^{i}\right),D_{5}=H_{4}(Ack\left\|M_{2}^{i}\right)$; finally, it sends ${VLP}_{2}=\{D_{4},D_{5},T_{2}\}$ to the vehicle, otherwise, it immediately terminates this session.

VLP-3: After receiving ${VLP}_{2}$, the vehicle calculates $({Ack}^{*}\left\|T_{2}\right)=D_{4}⊕H_{3}\left(M_{2}^{i}\right),\;{D_{5}}^{*}=H_{4}({Ack}^{*}\left\|M_{2}^{i}\right)$ and verifies whether ${D_{5}}^{*}$ equals $D_{5}$. If they are equal, the request succeeds, otherwise, the request fails.

### 4.4. Mutual Authentication and Session Key Establishment Phase

After completing the registration and login phases, the vehicle performs entity authentication based on the unloading policy.

#### 4.4.1. Offloading Scenario 1

When a vehicle needs to offload computational tasks to an edge server, the following authentication procedures are performed.

OS1-1: The vehicle first calculates ${Auth}_{V}^{i}=H_{1}({TID}_{V}^{i}\|{ID}_{V}^{i}\|{Res}_{i}\|X_{i})$, then generates a timestamp ${ts}_{1}$, and continues to compute $U_{1}=({TID}_{E}^{k}\|{ts}_{1})⊕H_{3}\left(M_{2}^{i}\right),{Int}_{1}=H_{1}({{Auth}_{V}^{i}\|U}_{1}\left\|{TID}_{V}^{i}\right\|{TID}_{E}^{k}\left\|{ts}_{1}\right),$ and finally sends ${Msg}_{1}^{V2I}=\{{Auth}_{V}^{i},U_{1},{TID}_{V}^{i},{Int}_{1},{ts}_{1}\}$ to the regional RSU.

OS1-2: Upon receiving ${Msg}_{1}^{V2I}$, the RSU first checks the timestamp’s freshness, then calculates $({{TID}_{E}^{k}}^{*}\left\|{{ts}_{1}}^{*}\right)=U_{1}⊕H_{3}\left(M_{2}^{i}\right),{{Int}_{1}}^{*}=H_{1}({Auth}_{V}^{i}{\|U}_{1}\left\|{TID}_{V}^{i}\right\|{{TID}_{E}^{k}}^{*}\left\|{{ts}_{1}}^{*}\right)$. It verifies whether ${{Int}_{1}}^{*}$ equals ${Int}_{1}$. If verification fails, the session is immediately terminated. Otherwise, it generates a random number $\beta ,\beta \in Z_{p}^{*}$ and continues calculating $M_{3}^{j}=\beta \times P,M_{4}^{j}=\beta \times {PK}_{ES}^{k}$. Then it generates a timestamp ${ts}_{2}$ and calculates $U_{2}=({TID}_{V}^{i}\left\|{ts}_{2}\right)⊕H_{3}\left(M_{4}^{j}\right),{Auth}_{R}^{j}=H_{1}\left({TID}_{R}^{j}\left\|{ID}_{RSU}^{j}\right\|Y_{j}\right),{Int}_{2}=H_{1}({M_{3}^{j}\|U}_{2}\|{Auth}_{R}^{j}\|{Auth}_{V}^{i}\|{TID}_{R}^{j}\|{TID}_{V}^{i}\|{ts}_{2})$, and finally sends ${Msg}_{2}^{V2I}=\{M_{3}^{j},U_{2},{Auth}_{V}^{i},{Auth}_{R}^{j},{TID}_{R}^{j},{ts}_{2}\}$ to the ES.

OS1-3: Upon receiving ${Msg}_{2}^{V2I}$, ES first verifies the timestamp’s freshness, then calculates $M_{4}^{j}=S_{E}^{k}{\;\times \;M}_{3}^{j},({{TID}_{V}^{i}}^{*}\left\|{{ts}_{2}}^{*}\right)=U_{2}⊕H_{3}\left(M_{4}^{j}\right),{{Int}_{2}}^{*}=H_{1}({M_{3}^{j}\|U}_{2}\|{Auth}_{R}^{j}\|{Auth}_{V}^{i}\|{TID}_{R}^{j}\|{{TID}_{V}^{i}}^{*}\|{{ts}_{2}}^{*})$, verifies whether ${{Int}_{2}}^{*}$ equals ${Int}_{2}$, and if they do not equal, immediately terminate the session. Otherwise, continue calculating ${Auth}_{E}^{k}=H_{1}=\left({ID}_{ES}^{k}\left\|{TID}_{E}^{k}\right\|Z_{k}\right)$, generate a timestamp ${ts}_{3}$, calculate $E_{1}={Enc}_{\left(Z_{k}\right)}\left\{{{TID}_{V}^{i}}^{*},{Auth}_{V}^{i},{TID}_{R}^{j},{Auth}_{R}^{j},{Auth}_{E}^{k},{ts}_{3}\right\},{Int}_{3}=H_{1}(E_{1}\|{TID}_{E}^{k}\|{Auth}_{E}^{k}{\|ts}_{3})$, and finally send ${Msg}_{3}^{V2I}=\{E_{1},{TID}_{E}^{k},{Int}_{3},{ts}_{3}\}$ to the CMC.

OS1-4: Upon receiving message ${Msg}_{3}^{V2I}$, CMC first verifies the timestamp’s freshness, then calculates ${ID}_{ES}^{k}={TID}_{E}^{k}⊕H_{1}(S\left\|{ID}_{CMC}\right),\;Z_{k}=H_{1}({ID}_{ES}^{k}\left\|S\right)$ and decrypts $\left\{{{TID}_{V}^{i}}^{**},\;{{Auth}_{V}^{i}}^{*},\;{{TID}_{R}^{j}}^{*},\;{{Auth}_{R}^{j}}^{*},\;{{Auth}_{E}^{k}}^{*},{{ts}_{3}}^{*}\;\right\}={Dec}_{\left(Z_{k}\right)}(E_{1})$. Then compute ${{Int}_{3}}^{*}=H_{1}(E_{1}\|{TID}_{E}^{k}\|{{Auth}_{E}^{k}}^{*}\|{{ts}_{3}}^{*})$, verify whether ${{Int}_{3}}^{*}$ equals ${Int}_{3}$, if not equal, immediately terminate the session. Otherwise, continue computing ${Auth}_{E}^{k}=H_{1}({TID}_{E}^{k}{\|ID}_{ES}^{k}\left\|Z_{k}\right),\;{ID}_{RSU}^{j}={{TID}_{R}^{j}}^{*}⊕H_{1}(S\left\|{ID}_{CMC}\right),\;{(ID}_{V}^{i}\|{PID}_{U}^{i})={{TID}_{V}^{i}}^{**}⊕H_{1}(S\left\|{ID}_{CMC}\right),\;X_{i}=H_{1}({ID}_{V}^{i}\left\|{PID}_{U}^{i}\right\|S\left\|{Res}_{i}\right),Y_{j}=H_{1}({ID}_{RSU}^{j}\left\|S\right),{Auth}_{V}^{i}=H_{1}({{TID}_{V}^{i}}^{*}\left\|{ID}_{V}^{i}\right\|{Res}_{i}\left\|X_{i}\right),\;{Auth}_{R}^{j}=H_{1}\left({{TID}_{R}^{j}}^{*}\left\|{ID}_{RSU}^{j}\right\|Y_{j}\right)$, verify whether ${Auth}_{E}^{k},\;{Auth}_{R}^{j},\;{Auth}_{V}^{i}$ are equal to ${{Auth}_{E}^{k}}^{*},\;{{Auth}_{R}^{j}}^{*},\;{{Auth}_{V}^{i}}^{*}$. If verification fails, immediately terminate the session. If verification succeeds, CMC generates a random number $\gamma ,\gamma \in Z_{p}^{*}$, selects a new set of “CRP”, and updates the temporary IDs for ES, RSU, and Vehicle: ${{TID}_{E}^{k}}^{new}=$ ${TID}_{E}^{k}⊕H_{1}(\gamma {\|Z}_{k}),\;{{TID}_{R}^{j}}^{new}=$ ${{TID}_{R}^{j}}^{*}⊕H_{1}(\gamma \|Y_{j}),\;{{TID}_{V}^{i}}^{new}=$ ${{TID}_{V}^{i}}^{**}⊕H_{1}\left(\gamma \left\|{{Res}_{i}}^{\prime }\right\|X_{i}\right)$, then continues calculating $U_{3}=Z_{k}⊕{{TID}_{R}^{j}}^{new},\;U_{4}=({{TID}_{E}^{k}}^{new}\|{{TID}_{V}^{i}}^{new})⊕({ID}_{RSU}^{j}\left\|Y_{j}\right),U_{5}=({{TID}_{R}^{j}}^{new}\left\|{{Cha}_{i}}^{\prime }\right)⊕{(X}_{i}\left\|{Res}_{i}\right)$. Next, generate a timestamp ${ts}_{4}$, calculate $\;U_{6}=\gamma ⊕H_{1}\left({ID}_{ES}^{k}\|{ts}_{4}\right),\;E_{2}={Enc}_{\left(Z_{k}⊕{ID}_{ES}^{k}\right)}\left\{U_{3},\;U_{4},\;U_{5},\;{U_{6},\;ts}_{4}\right\},\;{Int}_{4}=H_{1}(E_{2}\|{{TID}_{E}^{k}}^{new}\|{{TID}_{R}^{j}}^{new}\|{ts}_{4})$, and finally send ${Msg}_{4}^{V2I}=\{E_{2},\;{Int}_{4},\;{ts}_{4}\}$ to the ES.

OS1-5: Upon receiving message ${Msg}_{4}^{V2I}$, ES first verifies the timestamp’s freshness, then decrypts $\left\{{U_{3}}^{*},{U_{4}}^{*},{U_{5}}^{*},{U_{6}}^{*},{{ts}_{4}}^{*}\right\}={Dec}_{\left(Z_{k}⊕{ID}_{ES}^{k}\right)}(E_{2})$, computes $\gamma ={U_{6}}^{*}⊕H_{1}\left({ID}_{ES}^{k}\|{{ts}_{4}}^{*}\right),{{TID}_{E}^{k}}^{new}=$ ${TID}_{E}^{k}⊕H_{1}\left(\gamma {\|Z}_{k}\right),{{TID}_{R}^{j}}^{new}=Z_{k}⊕{U_{3}}^{*},{{Int}_{4}}^{*}=H_{1}(E_{2}\|{{TID}_{E}^{k}}^{new}\|{{TID}_{R}^{j}}^{new}\|{{ts}_{4}}^{*})$, and verifies whether ${{Int}_{4}}^{*}$ equals ${Int}_{4}$. If verification fails, immediately terminate the session. Otherwise, continue calculating ${SK}_{E-R}^{k-j}=H_{4}\left({{TID}_{E}^{k}}^{\;new}\|{{TID}_{R}^{j}}^{\;new}\|M_{4}^{j}\right)$, and generate a timestamp ${ts}_{5}$, calculate $U_{7}=H_{3}\left(M_{4}^{j}\|{ts}_{5}\right)⊕\gamma ,{Int}_{5}=H_{1}({U_{4}}^{*}\|{U_{5}}^{*}\|U_{7}\|{SK}_{E-R}^{k-j}{\|\gamma \|ts}_{5})$, and finally send ${Msg}_{5}^{V2I}=\{{U_{4}}^{*},{U_{5}}^{*},U_{7},{Int}_{5},{ts}_{5}\}$ to the RSU.

OS1-6: Upon receiving message ${Msg}_{5}^{V2I}$, RSU first verifies the timestamp’s freshness, then calculates $\gamma =U_{7}⊕H_{3}\left(M_{4}^{j}\|{ts}_{5}\right),{{TID}_{R}^{j}}^{new}={{TID}_{R}^{j}}^{*}⊕H_{1}(\gamma \|Y_{j}),({{TID}_{E}^{k}}^{new}\|{{TID}_{V}^{i}}^{new})={U_{4}}^{*}⊕({ID}_{RSU}^{j}\|Y_{j}),{SK}_{R-E}^{j-k}=H_{4}\left({{TID}_{E}^{k}}^{\;new}\|{{TID}_{R}^{j}}^{\;new}\|M_{4}^{j}\right)$, ${{Int}_{5}}^{*}=H_{1}({U_{4}}^{*}\|{U_{5}}^{*}\|U_{7}{\|SK}_{R-E}^{j-k}\|{\gamma \|ts}_{5})$, and verifies whether ${{Int}_{5}}^{*}$ equals ${Int}_{5}$. If verification fails, immediately terminate the session. Otherwise, generates a timestamp ${ts}_{6}$, calculate $C_{1}=H_{1}({SK}_{R-E}^{j-k}\|{ts}_{6})$; next, send ${Msg}_{6}^{V2I}=\{C_{1},{ts}_{6}\}$ to ES.

OS1-7: Upon receiving the message ${Msg}_{6}^{V2I}$, ES first verifies the timestamp’s freshness, then calculates ${C_{1}}^{*}=H_{1}({SK}_{R-E}^{j-k}\|{ts}_{6})$, verifies whether ${C_{1}}^{*}$ equals $C_{1}$, and if the verification succeeds, it indicates that the session key between the ES and the RSU has been correctly established. Otherwise, the session is immediately terminated.

OS1-8: After sending the ${Msg}_{6}$, the RSU calculates ${SK}_{R-V}^{j-i}=H_{4}({{TID}_{R}^{j}}^{new}\|{{TID}_{V}^{i}}^{new}\|M_{2}^{i})$, then generates a timestamp ${ts}_{7}$, continues to calculate $U_{8}=\gamma ⊕H_{4}(M_{2}^{i}\left\|{ts}_{7}\right),{Int}_{6}=H_{1}({U_{5}}^{*}\|U_{8}\|\gamma {\|SK}_{R-V}^{j-i}\|{ts}_{7})$, and finally sends ${Msg}_{7}^{V2I}=\{{U_{5}}^{*},U_{8},{Int}_{6},{ts}_{7}\}$ to the vehicle.

OS1-9: Upon receiving the message ${Msg}_{7}^{V2I}$, the vehicle first verifies the timestamp’s freshness, then calculates $\gamma =U_{8}⊕H_{4}(M_{2}^{i}\left\|{ts}_{7}\right)$, $({{TID}_{R}^{j}}^{new}\left\|{{Cha}_{i}}^{\prime }\right)={U_{5}}^{*}⊕{(X}_{i}\left\|{Res}_{i}\right),PUF\left({{Cha}_{i}}^{\prime }\right)={{Res}_{i}}^{\prime },{{TID}_{V}^{i}}^{new}={TID}_{V}^{i}⊕H_{1}\left(\gamma \left\|{{Res}_{i}}^{\prime }\right\|X_{i}\right)$, ${SK}_{V-R}^{i-j}=H_{4}\left({{TID}_{R}^{j}}^{\;new}\|{{TID}_{V}^{i}}^{\;new}\|M_{2}^{i}\right)$, and ${{Int}_{6}}^{*}=H_{1}({U_{5}}^{*}\|U_{8}\|{SK}_{V-R}^{i-j}\|\gamma \|{ts}_{7})$, and verifies whether ${{Int}_{6}}^{*}$ equals ${Int}_{6}$. If verification fails, immediately terminate the session. Otherwise, it generates a timestamp ${ts}_{8}$ and calculates $C_{2}=H_{1}({SK}_{V-R}^{i-j}\|{ts}_{8})$; finally, the vehicle send ${Msg}_{8}^{V2I}=\left\{C_{2},{ts}_{8}\right\}$ to the RSU.

OS1-10: Upon receiving the message ${Msg}_{8}^{V2I}$, RSU first verifies the timestamp’s freshness, then calculates ${C_{2}}^{*}=H_{1}({SK}_{R-V}^{k-i}\|{ts}_{6})$, verifies whether ${C_{2}}^{*}$ equals $C_{2}$, and if the verification succeeds, it indicates that the session key between the RSU and the vehicle has been correctly established. Otherwise, the session is immediately terminated.

**Figure 2:**
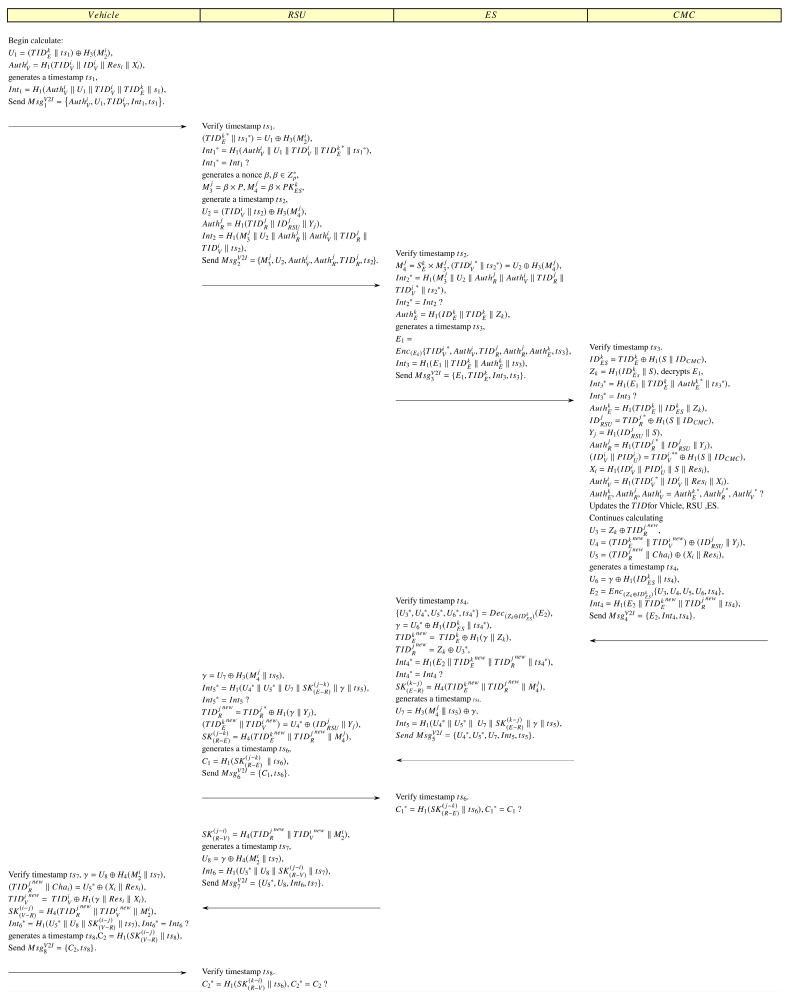
Authentication process for offloading scenario 1.

Figure 2 illustrates a specific process of offloading scenario 1.

#### 4.4.2. Offloading Scenario 2

**Figure 3:**
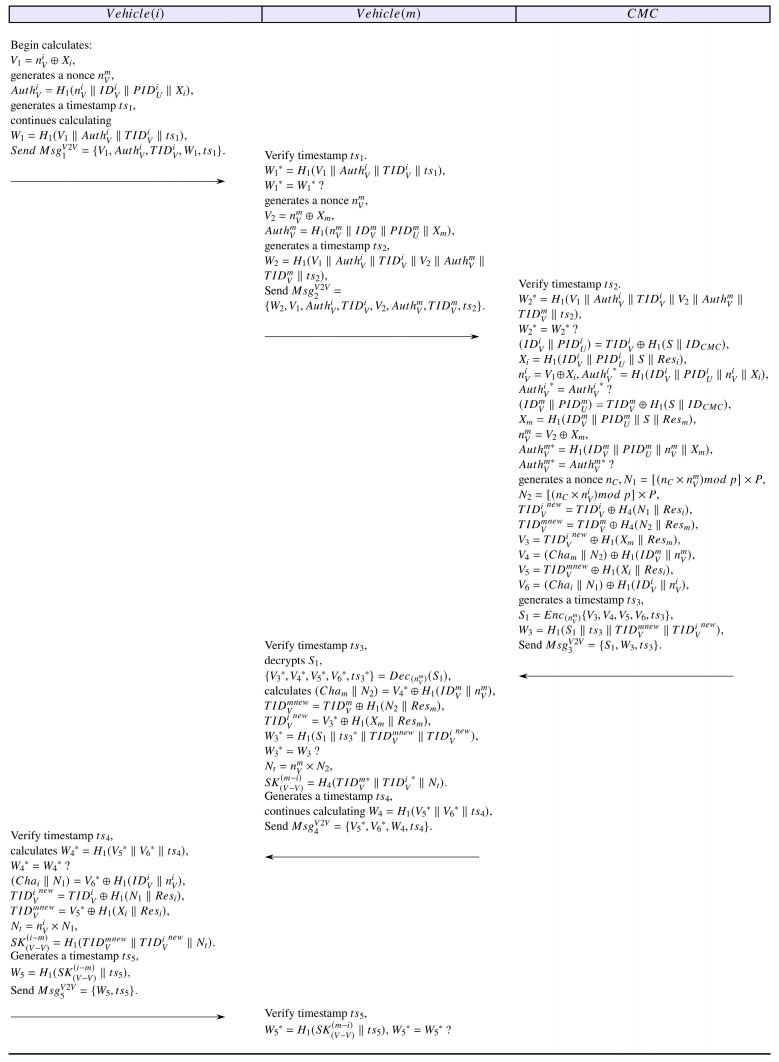
Authentication process for offloading scenario 2.

When a vehicle needs to offload computational tasks to an auxiliary vehicle, the following authentication process will be executed. Figure 3 illustrates a specific process of offloading scenario 2.

OS2-1: The vehicle requesting offloading first generates a random number $n_{V}^{i}$, then calculates $V_{1}=n_{V}^{i}⊕X_{i},\;{Auth}_{V}^{i}=H_{1}(n_{V}^{i}\|{ID}_{V}^{i}\|{PID}_{U}^{i}\|X_{i})$; next, it generates a timestamp ${ts}_{1}$, continues calculating $W_{1}=H_{1}(V_{1}\|{Auth}_{V}^{i}\|{TID}_{V}^{i}\|{ts}_{1})$; finally, it sends ${Msg}_{1}^{V2V}=\{V_{1},\;{Auth}_{V}^{i},\;{TID}_{V}^{i},W_{1},\;{ts}_{1}\}$, to the auxiliary vehicle.

OS2-2: Upon receiving message ${Msg}_{1}^{V2V}$, the auxiliary vehicle first verifies the timestamp’s freshness and calculates ${W_{1}}^{*}=H_{1}(V_{1}\|{Auth}_{V}^{i}\|{TID}_{V}^{i}\|{ts}_{1})$, and verifies whether ${W_{1}}^{*}$ equals $W_{1}$. If verification fails, it immediately terminates the session. Otherwise, he generates a random number $n_{V}^{m}$ and continues calculating $V_{2}=n_{V}^{m}⊕X_{m},\;{Auth}_{V}^{m}=H_{1}(n_{V}^{m}\|{ID}_{V}^{m}\|{PID}_{U}^{m}\|X_{m})$, then generates a timestamp ${ts}_{2}$, continues calculating $W_{2}=H_{1}\left(V_{1}\right\|{Auth}_{V}^{i}\left\|\;{TID}_{V}^{i}\right\|V_{2}\left\|\;{Auth}_{V}^{m}\right\|{TID}_{V}^{m}\left\|\;{ts}_{2}\right),$ and sends ${Msg}_{2}^{V2V}=\{{W_{2},\;V}_{1},\;{Auth}_{V}^{i},\;{TID}_{V}^{i},V_{2},\;{Auth}_{V}^{m},\;{TID}_{V}^{m},\;{ts}_{2}\}$ to the CMC.

OS2-3: Upon receiving message ${Msg}_{2}^{V2V}$, CMC first verifies the timestamp’s freshness and computes ${W_{2}}^{*}=H_{1}(V_{1}\|{Auth}_{V}^{i}\|{TID}_{V}^{i}\|V_{2}\|{Auth}_{V}^{m}\|{TID}_{V}^{m}\|{ts}_{2})$, then validates whether ${W_{2}}^{*}$ equals $W_{2}$. If they do not equal, the session is immediately terminated. Otherwise, it proceeds to calculate $\left({ID}_{V}^{i}\|{PID}_{U}^{i})={TID}_{V}^{i}⊕H_{1}(S\left\|{ID}_{CMC}\right),X_{i}=H_{1}({ID}_{V}^{i}\|{PID}_{U}^{i}\|S\left\|{Res}_{i}\right),{{n_{V}^{i}=V_{1}⊕X_{i},Auth}_{V}^{i}}^{*}=H_{1}({ID}_{V}^{i}\|{PID}_{U}^{i}\|n_{V}^{i}\|X_{i}\right)$; CMC verifies whether ${{Auth}_{V}^{i}}^{*}$ equals ${Auth}_{V}^{i}$. If they are equal, it indicates that CMC has successfully verified the requesting vehicle. Otherwise, the verification fails. CMC continues to calculate $\left({ID}_{V}^{m}\|{PID}_{U}^{m})={TID}_{V}^{m}⊕H_{1}(S\left\|{ID}_{CMC}\right),X_{m}=H_{1}({ID}_{V}^{m}\left\|{PID}_{U}^{m}\right\|S\left\|{Res}_{m}\right),{{n_{V}^{m}=V_{2}⊕X_{m},Auth}_{V}^{m}}^{*}=H_{1}({ID}_{V}^{m}\|{PID}_{U}^{m}\|n_{V}^{m}\|X_{m}\right)$, then verifies whether ${{Auth}_{V}^{m}}^{*}$ equals ${Auth}_{V}^{m}$. Similarly, if they are equal, it indicates that CMC has successfully verified the auxiliary vehicle. Subsequently, CMC generates a random number $n_{C}$, selects a new set of “CRP” and updates the temporary IDs for the requesting vehicle and the auxiliary vehicle: ${{N_{1}=\left[\left(n_{C}\times n_{V}^{m}\right)mod\;p\right]\times P,N_{2}=[\left(n_{C}\times n_{V}^{i}\right)mod\;p]\times P,TID}_{V}^{i}}^{new}={TID}_{V}^{i}⊕H_{4}(N_{1}\left\|{{Res}_{i}}^{\prime }\right),{{TID}_{V}^{m}}^{new}={TID}_{V}^{m}⊕H_{4}(N_{2}\left\|{{Res}_{m}}^{\prime }\right)$, then, it calculates $V_{3}={{TID}_{V}^{i}}^{new}⊕H_{1}{(X}_{m}\|{{Res}_{m}}^{\prime }),V_{4}=({{Cha}_{m}}^{\prime }\|N_{2})⊕H_{1}({ID}_{V}^{m}\left\|n_{V}^{m}\right),V_{5}={{TID}_{V}^{m}}^{new}⊕{H_{1}(X}_{i}\|{{Res}_{i}}^{\prime }),V_{6}=({{Cha}_{i}}^{\prime }\|N_{1})⊕H_{1}({ID}_{V}^{i}\|n_{V}^{i})$; next, it generates a timestamp ${ts}_{3}$ and an encrypted message $S_{1}={Enc}_{\left(n_{V}^{m}\right)}{\{V}_{3},V_{4},V_{5},V_{6},{ts}_{3}\}$, calculates $W_{3}=H_{1}(S_{1}\left\|{ts}_{3}\right\|{{TID}_{V}^{m}}^{new}\|{{TID}_{V}^{i}}^{new}),$ and finally sends ${Msg}_{3}^{V2V}=\{S_{1},W_{3},{ts}_{3}\}$ to the auxiliary vehicle.

OS2-4: Upon receiving message ${Msg}_{3}^{V2V}$, the auxiliary vehicle first verifies the timestamp’s freshness, then decrypts $\left\{{V_{3}}^{*},{V_{4}}^{*},{V_{5}}^{*},{V_{6}}^{*},{{ts}_{3}}^{*}\right\}={Dec}_{\left(n_{V}^{m}\right)}(S_{1})$, calculates $({{Cha}_{m}}^{\prime }\left\|N_{2}\right)={{V_{4}}^{*}⊕H}_{1}({ID}_{V}^{m}\left\|n_{V}^{m}\right),{{TID}_{V}^{m}}^{new}={TID}_{V}^{m}⊕H_{1}(N_{2}\left\|{{Res}_{m}}^{\prime }\right),{{TID}_{V}^{i}}^{new}={V_{3}}^{*}⊕H_{1}{(X}_{m}\left\|{{Res}_{m}}^{\prime }\right),{W_{3}}^{*}=H_{1}(S_{1}\left\|{{ts}_{3}}^{*}\right\|{{TID}_{V}^{m}}^{new}\left\|{{TID}_{V}^{i}}^{\;new}\right)$, and validates whether ${W_{3}}^{*}$ equals $W_{3}$. If they do not equal, the session is immediately terminated. Otherwise, it proceeds to calculate $N_{t}=n_{V}^{m}\times N_{2}$, after that, it establishes the session key ${SK}_{V-V}^{m-i}=H_{4}({{TID}_{V}^{m}}^{*}\|{{TID}_{V}^{i}}^{*}\|N_{t})$. Then, it generates a timestamp ${ts}_{4}$, continues to calculate $W_{4}=H_{1}({V_{5}}^{*}\|{V_{6}}^{*}\|{ts}_{4})$, and finally sends ${Msg}_{4}^{V2V}=\{{V_{5}}^{*},{V_{6}}^{*},W_{4},{ts}_{4}\}$ to the requesting vehicle.

OS2-5: Upon receiving the message ${Msg}_{4}^{V2V}$, the requesting vehicle first verifies the timestamp’s freshness, then computes ${W_{4}}^{*}=H_{1}({V_{5}}^{*}\|\;{V_{6}}^{*}\|{ts}_{4})$, verifying whether ${W_{4}}^{*}$ equals $W_{4}$. If unequal, the session is immediately terminated, otherwise, it continues computing $({{Cha}_{i}}^{\prime }\left\|N_{1}\right)={{V_{6}}^{*}⊕H}_{1}({ID}_{V}^{i}\left\|n_{V}^{i}\right),\;{{TID}_{V}^{i}}^{new}={TID}_{V}^{i}⊕H_{1}(N_{1}\left\|{{Res}_{i}}^{\prime }\right),\;{{TID}_{V}^{m}}^{new}={V_{5}}^{*}⊕H_{1}{(X}_{i}\left\|{{Res}_{i}}^{\prime }\right),\;N_{t}=n_{V}^{i}\times N_{1}$. Similarly, it establishes the session key ${SK}_{V-V}^{i-m}=H_{1}({{TID}_{V}^{m}}^{new}\|{{TID}_{V}^{i}}^{new}\|N_{t})$. Next, it generates a timestamp ${ts}_{5}$ and continues calculating $W_{5}=H_{1}({SK}_{V-V}^{i-m}\|{ts}_{5})$, and finally, it sends ${Msg}_{5}^{V2V}=\{W_{5},{ts}_{5}\}$ to the auxiliary vehicle.

OS2-6: Upon receiving the message ${Msg}_{5}^{V2V}$, the auxiliary vehicle first verifies the timestamp’s freshness, then calculates ${W_{5}}^{*}=H_{1}({SK}_{V-V}^{m-i}\|{ts}_{5})$, verifies whether ${W_{5}}^{*}$ equals $W_{5}$, and if the verification succeeds, it indicates that the session key between the requesting vehicle and the auxiliary vehicle has been correctly established. Otherwise, the session is immediately terminated.

## 5. Formal Analysis and Informal Analysis

In this section, we perform both formal and informal security analyses on the proposed scheme to evaluate its security.

### 5.1. Formal Analysis Using BAN Logic

BAN logic is a formal logical tool for analyzing and verifying protocol security [36]. It transforms protocol security into provable problems through belief reasoning [37]. The primary concepts and rules of BAN logic are shown in Table 3 and Table 4.

Based on the concepts and rules of the BAN logic outlined above, the security and correctness of the solution are verified through the following specific reasoning process (we take OS1 as an example.):

1. The goals we need to prove:

Goal 1: $V_{i}|\equiv (V_{i}\overset{SK_{ij}}{↔}RSU_{j})$ Goal 2: $RSU_{j}|\equiv (V_{i}\overset{SK_{ij}}{↔}RSU_{i})$

Goal 3: $V_{i}|\equiv RSU_{j}|\equiv (V_{i}\overset{SK_{ij}}{↔}RSU_{j})$ Goal 4: $RSU_{j}|\equiv V_{i}|\equiv (V_{i}\overset{SK_{ij}}{↔}RSU_{j})$

Goal 5: $RSU_{j}|\equiv (RSU_{j}\overset{SK_{jk}}{↔}ES_{k})$ Goal 6: $ES_{k}|\equiv (RSU_{j}\overset{SK_{jk}}{↔}ES_{k})$

Goal 7: $RSU_{j}|\equiv ES_{k}|\equiv (RSU_{j}\overset{SK_{jk}}{↔}ES_{k})$

Goal 8: $ES_{k}|\equiv RSU_{j}|\equiv (RSU_{j}\overset{SK_{jk}}{↔}ES_{k})$

2. Initial assumptions:

A1: $V_{i}|\equiv (V_{i}\overset{M_{2}^{i},X_{i},Res_{i}}{↔}RSU_{j})$ A2: $RSU_{j}|\equiv (V_{i}\overset{M_{2}^{i},X_{i},Res_{i}}{↔}RSU_{j})$

A3: $RSU_{j}|\equiv \text{\#}(ts_{2})$ A4: $ES_{k}|\equiv (RSU_{j}\overset{M_{4}^{j},Y_{j}}{↔}ES_{k})$

A5: $RSU_{j}|\equiv (RSU_{j}\overset{M_{4}^{j},Y_{j}}{↔}ES_{k})$ A6: $V_{i}|\equiv RSU_{j}\Rightarrow (V_{i}\overset{SK_{ij}}{↔}RSU_{j})$

A7: $RSU_{j}|\equiv V_{i}\Rightarrow (V_{i}\overset{SK_{ij}}{↔}RSU_{j})$ A8: $ES_{k}|\equiv \#(ts_{1})$

A9: $ES_{k}|\equiv (ES_{k}\overset{Z_{k},ID_{ES}^{k}}{↔}RSU_{j})$ A10: $V_{i}|\equiv \#(ts_{3})$

A11: $RSU|\equiv \#(ts_{5})$ A13: $ES_{k}|\equiv \#(ts_{4})$

A14: $RSU_{j}|\equiv ES_{k}\Rightarrow (RSU_{j}\overset{SK_{jk}}{↔}ES_{k})$

A15: $ES_{k}|\equiv RSU_{j}\Rightarrow (RSU_{j}\overset{SK_{jk}}{↔}ES_{k})$

3. Idealized scheme:

The idealized form of the BAN logic for the proposed scheme is as follows:

$$\begin{array}{l} M_{1}=({\left\{TID_{R}^{j\;new},\gamma \right\}}_{\left(Z_{k},ID_{ES}^{k}\right)},ts_{1},{\left\{TID_{E}^{k\;new}\right\}}_{\left(Z_{k}\right)})\\ M_{2}=({\left\{\gamma \right\}}_{\left(M_{4}^{j}\right)},{\left\{TID_{E}^{k\;new},TID_{V}^{i\;new}\right\}}_{\left(Y_{j}\right)},{\left\{TID_{R}^{j\;new}\right\}}_{\left(\gamma ,Y_{j}\right)},ts_{6})\\ M_{3}=({\left\{\gamma \right\}}_{\left(M_{2}^{i}\right)},{\left\{TID_{R}^{j\;new}\right\}}_{\left(X_{i},Res_{i}\right)},{\left\{TID_{V}^{i\;new}\right\}}_{\left(X_{i}\right)},ts_{6})\\ M_{4}=({\left\{SK_{jk}\right\}}_{\left(M_{4}^{j}\right)},ts_{7})\\ M_{5}=(SK_{ij},ts_{8}) \end{array}$$

4. Process of logical reasoning:

P1: From $M_{1}$, we obtain

$$ES_{k}⊲({\left\{TID_{R}^{j\;new},\gamma \right\}}_{\left(Z_{k},ID_{ES}^{k}\right)},ts_{1},{\left\{TID_{E}^{k\;new}\right\}}_{\left(Z_{k}\right)})$$

P2: From P1, A9, and by applying the R1, we deduce

$$ES_{k}|\equiv CMC|∼(TID_{R}^{j\;new},TID_{E}^{k\;new},\gamma )$$

P3: From P2, A8, and by applying the R4, we deduce

$$ES_{k}|\equiv \#(TID_{R}^{j\;new},TID_{E}^{k\;new},\gamma )$$

P4: From $M_{2}$, we obtain

$$RSU_{j}⊲({\left\{\gamma \right\}}_{\left(M_{4}^{j}\right)},{\left\{TID_{E}^{knew},TID_{V}^{i\;new}\right\}}_{\left(Y_{j}\right)},{\left\{TID_{R}^{j\;new}\right\}}_{\left(\gamma ,Y_{j}\right)},ts_{2})$$

P5: From P4, A5, and by applying the R1, we deduce

$$RSU_{j}|\equiv ES_{k}|∼(TID_{E}^{k\;new},TID_{V}^{i\;new},\gamma ,TID_{R}^{j\;new})$$

P6: From P5, A3, and by applying the R4, we deduce

$$RSU_{j}|\equiv \#(TID_{E}^{k\;new},TID_{V}^{i\;new},\gamma ,TID_{R}^{j\;new})$$

P7: From P5, P6, and by applying the R2, we deduce

$$RSU_{j}|\equiv ES_{k}|\equiv (TID_{E}^{k\;new},TID_{R}^{j\;new})$$

P8: From Msg6, we obtain

$$V_{i}⊲({\left\{\gamma \right\}}_{\left(M_{2}^{i}\right)},{\left\{TID_{R}^{j\;new}\right\}}_{\left(X_{i},Res_{i}\right)},{\left\{TID_{V}^{i\;new}\right\}}_{\left(X_{i}\right)},ts_{3})$$

P9: From P8, A1, and by applying the R1, we deduce

$$V_{i}|\equiv RSU_{j}|∼(\gamma ,TID_{R}^{j\;new},TID_{V}^{i\;new})$$

P10: From P9, A10, and by applying the R4, we deduce

$$V_{i}\#(\gamma ,TID_{R}^{j\;new},TID_{V}^{i\;new})$$

P11: From Msg, we obtain

$$ES_{k}⊲({\left\{SK_{jk}\right\}}_{\left(M_{4}^{j}\right)},ts_{4})$$

P12: From P11, A4, and by applying the R1, we deduce

$$ES_{k}|\equiv RSU_{j}|∼(SK_{jk})$$

P13: From P12, A12, and by applying the R4, we deduce

$$ES_{k}|\equiv \#(SK_{jk})$$

P14: From Msg, we obtain

$$RSU_{j}⊲(SK_{ij},ts_{5})$$

P15: From P14, A5, and by applying the R1, we deduce

$$RSU_{j}|\equiv ES_{k}|∼(SK_{ij})$$

P16: From P15, A13, and by applying the R4, we deduce

$$RSU_{j}|\equiv \#(SK_{ij})$$

P17: From P9, P10, A1 and by applying the R2, we deduce

$$V_{i}|\equiv RSU_{j}|\equiv (V_{i}\overset{SK_{ij}}{↔}RSU_{j})\;(\mathrm{Goal}\;3)$$

P18: From P17, A6 and by applying the R3, we deduce

$$V_{i}|\equiv (V_{i}\overset{SK_{ij}}{↔}RSU_{j})\;(\mathrm{Goal}\;1)$$

P19: From P12, P13, A9 and by applying the R2, we deduce

$$ES_{k}|\equiv RSU_{j}|\equiv (RSU_{j}\overset{SK_{jk}}{↔}ES_{k})\;(\mathrm{Goal}\;8)$$

P20: From P19, A15 and by applying the R3, we deduce

$$ES_{k}|\equiv (RSU_{j}\overset{SK_{jk}}{↔}ES_{k})\;(\mathrm{Goal}\;6)$$

P21: From P7 and A5, we deduce

$$RSU_{j}|\equiv ES_{k}|\equiv (RSU_{j}\overset{SK_{jk}}{↔}ES_{k})\;(\mathrm{Goal}\;7)$$

P22: From P2, A14, and by applying the R3, we deduce

$$RSU_{j}|\equiv (RSU_{j}\overset{SK_{jk}}{↔}ES_{k})\;(\mathrm{Goal}\;5)$$

P23: From P15, P16, and by applying the R2, we deduce

$$RSU_{j}|\equiv V_{i}|\equiv (V_{i}\overset{SK_{ij}}{↔}RSU_{j})\;(\mathrm{Goal}\;4)$$

P24: From P23, A, and by applying the R2, we deduce

$$RSU_{j}|\equiv (V_{i}\overset{SK_{ij}}{↔}RSU_{j})\;(\mathrm{Goal}\;2)$$

The above reasoning achieves all security goals, showing that the proposed scheme passes the BAN logical verification analysis.

### 5.2. Formal Analysis Using ROR Oracle Model

In this section, we employ the Real-Or-Random (ROR) model [38] to evaluate the semantic security of the proposed scheme. The participating entity types include a vehicle (V), a roadside unit (RSU), an edge server (ES), and a cloud management center (CMC). Their respective instances are denoted as $\Pi_{V}^{i},\;\Pi_{RSU}^{j},\;\Pi_{ES}^{k},\;\Pi_{CMC}^{l}$.

In the model, the capabilities of adversary A are defined through a set of queries. The specific queries are detailed in Table 5.

- Partnership: If two instances are partners, they must satisfy the following conditions:

(1) They are in the same session.
(2) They can send and receive corresponding messages to and from each other.
(3) Other instances besides them will not accept the session keys established between them.

- Freshness: If $\Pi_{\Delta }^{ο}$ is deemed fresh, it must satisfy the following conditions:

(1) $\Pi_{\Delta }^{ο}$ is in the accepted state. It indicates that $\Pi_{\Delta }^{ο}$ has successfully completed the session.
(2) A has never called Send query for $\Pi_{\Delta }^{ο}$.
(3) $\Pi_{\Delta }^{ο}$ and its partners have not been queried by A using Reveal().

- Session Key Semantic Security: The advantage of adversary A is a precise definition quantifying the probability of A’s attack succeeding beyond random guessing. If adversary A performs a Test() query on a fresh instance and successfully returns the correct guess b’ = b, this indicates A’s success. Advantage A is defined as $Adv_{A}=2|P[b^{\prime }=b]-1/2|$. If $Adv_{A}$ is negligible for any polynomial-time adversary A, then our proposed scheme is secure.

**Theorem** **1.** *Suppose there exists an adversary*A *running in polynomial time whose goal is to undermine the semantic security of session keys in the proposed scheme.*A*’s winning advantage in the ROR security game is defined as*

$$\begin{array}{ll} Adv_{A} & \leq \frac{\sum_{i=1}^{4}q_{H_{i}}^{2}}{2^{\left(l_{1}+1\right)}}+\frac{q_{puf}^{2}}{2^{\left(l_{2}+1\right)}}+\frac{3{\left(q_{S}^{OS\delta }+q_{E}^{OS\delta }\right)}^{2}}{2p}+\frac{{\left(q_{S}^{OS\delta }+q_{E}^{OS\delta }\right)}^{3}}{6p^{2}}+\frac{q_{S}^{2}}{2^{l_{1}}}\\ & +Max\{C_{Z}\times q_{S}^{s},\frac{q_{S}}{2^{u}}\}+Adv_{A}^{ECDHP} \end{array}$$

We prove this theorem through the following game.

**Proof** **of** **Theorem** **1.** Game 0: Game 0 is the initial game, simulating the protocol’s execution in a real-world environment. A can initiate all oracle queries (Send(), Execute(), Reveal(), Corrupt()) to C, who will return the corresponding responses. Game 0 defines A’s attack capabilities within the actual protocol. The adversary’s advantage is defined as

(1) $$Adv_{A}=|2\times Adv_{A,Game0}-1|$$

Game 1: In Game 1, a hash oracle query $H_{i}$ is defined. C maintains a list, List($H_{i}$), of Hash oracle queries. If A invokes a hash oracle query on message m, C queries List($H_{i}$). If the list contains a matching $\langle m,H_{i}(m)\rangle$ entry, C directly returns it to A. If no matching entry is found, C selects a uniform random output value $H_{i}(m)$, returns this randomly generated hash value to A, and adds < m, $H_{i}(m)$ > to List($H_{i}$). Game 1 is identical to Game 0, so we have

(2) $$|Adv_{A,Game1}-Adv_{A,Game0}|=0$$

Game 2: Simulates all oracle queries from Game 1 and, building upon Game 1, defines three types of collisions using the birthday paradox [39]:

(1) Probability of hash function collisions:
  $$P_{H}=\frac{\sum_{i=1}^{4}q_{H_{i}}^{2}}{2^{\left(l_{1}+1\right)}}$$
(2) Probability of PUF collision:
  $$P_{puf}=\frac{q_{puf}^{2}}{2^{(l_{2}+1)}}$$

The probability of a collision between random numbers in OS1 and OS2 is

$$P_{nonce}=\frac{3{\left(q_{S}^{OS\delta }+q_{E}^{OS\delta }\right)}^{2}}{2p}+\frac{{\left(q_{S}^{OS\delta }+q_{E}^{OS\delta }\right)}^{3}}{6p^{2}}(\delta =1,2)$$

Therefore,

(3) $$\begin{array}{ll} \left|Adv_{A,Game2}-Adv_{A,Game1}\right| & \leq \frac{\sum_{i=1}^{4}q_{H_{i}}^{2}}{2^{\left(l_{1}+1\right)}}+\frac{q_{puf}^{2}}{2^{\left(l_{2}+1\right)}}+\frac{3{\left(q_{S}^{OS\delta }+q_{E}^{OS\delta }\right)}^{2}}{2p}\\ & +\frac{{\left(q_{S}^{OS\delta }+q_{E}^{OS\delta }\right)}^{3}}{6p^{2}}(\delta =1,2) \end{array}$$

Game 3: Simulates all queries from Game 2, but in this game, adversary A directly obtains certain authentication-related values without invoking the hash oracle query. Therefore, in this game, we can conclude that

(4) $$|Adv_{A,Game3}-Adv_{A,Game2}|\leq \frac{q_{S}^{2}}{2^{l_{1}}}$$

Game 4: Simulates all queries from Game 3. In this game, we specifically consider the scenario where A invokes the Corrupt() query to query C, and C returns the corresponding response to A. A can extract $\left\{A_{i},\;B_{i},\;C_{i},\;{TID}_{V}^{i}\right\}$ from the OBU. A attempts to compute $X_{i}$, but lacks the correct ${PID}_{U}^{i}$ and ${PPW}_{U}^{i}$, making $X_{i}$ calculation infeasible. Furthermore, A cannot construct a valid ${TID}_{V}^{i}$. Applying Zipf’s Law [40], we conclude that $C_{Z},q_{S}^{s}$ are two core parameters in Zipf’s Law:

(5) $$\left|Adv_{A,Game4}-Adv_{A,Game3}|\leq Max\{C_{Z}\times q_{S}^{s},\frac{q_{S}}{2^{u}}\right\}$$

Game 5: In this game, we consider the problem that A must solve to construct the correct session key. For the session key SK to be established, A must solve the ECDH problem. Therefore, we conclude that

(6) $$|Adv_{A,Game5}-Adv_{A,Game4}|\leq Adv_{A}^{ECDHP}$$

In this game, A can only make random guesses. If A initiates any other queries, the game terminates. Therefore, A holds no advantage in this game, and we conclude that

(7) $$Adv_{A,Game5}=\frac{1}{2}$$

By combining (1), (2) and (7), we can obtain

(8) $$\begin{array}{ll} \frac{1}{2}Adv_{A} & =|Adv_{A,Game0}-\frac{1}{2}|=|Adv_{A,Game1}-\frac{1}{2}|\\ & =|\;Adv_{A,Game0}-Adv_{A,Game5}| \end{array}$$

Then, through (3)–(6) and (8), we obtain

$$\begin{array}{ll} \frac{1}{2}Adv_{A} & =|Adv_{A,Game1}-Adv_{A,Game4}|\\ & \leq |Adv_{A,Game1}-Adv_{A,Game2}|\\ & +|Adv_{A,Game2}-Adv_{A,Game3}|\\ & +|Adv_{A,Game3}-Adv_{A,Game4}|\\ & +|Adv_{A,Game4}-Adv_{A,Game5}|\\ & \leq \frac{\sum_{i=1}^{4}q_{H_{i}}^{2}}{2^{\left(l_{1}+1\right)}}+\frac{q_{puf}^{2}}{2^{\left(l_{2}+1\right)}}+\frac{3{\left(q_{S}^{OS\delta }+q_{E}^{OS\delta }\right)}^{2}}{2p}+\frac{{\left(q_{S}^{OS\delta }+q_{E}^{OS\delta }\right)}^{3}}{6p^{2}}\\ & \;+\frac{q_{S}^{2}}{2^{l_{1}}}+Max\{C_{Z}\times q_{S}^{s},\frac{q_{S}}{2^{u}}\}+Adv_{A}^{ECDHP} \end{array}$$

Thus, we obtain Theorem, which proves the security of our scheme under the ROR model. □

### 5.3. Formal Analysis Using AVISPA

We employed the formal verification tool AVISPA to conduct security analysis on our designed protocol, which can demonstrate whether our protocol possesses fundamental security properties and the ability to defend against critical attacks. AVISPA [41] stands for “Automated Verification of Internet Security Protocols and Applications.” It provides an advanced protocol specification language called “HLPSL [42].” We employ HLPSL to describe our security protocols and use the “HLPSL2IF” translator to convert the HLPSL specification into an “Intermediate Format (IF)” specification. IF is a lower-level language specification than HLPSL, enabling it to be directly read by the backend modules of AVISPA tools. AVISPA tools comprise four backend modules [41]: The “On-the-Fly Model-Checker” (OFMC), which efficiently verifies protocol security in bounded sessions through dynamic symbolic (Dolev–Yao) model checking; The CL-based Model-Checker (CL-AtSe) converts the security specifications of a protocol into a set of logical constraints, such as message freshness and consistency. By solving these logical constraints, it identifies various potential attacks against the protocol, including replay attacks and key leakage; the SAT-based Model-Checker (SATMC) transforms protocol security verification problems into satisfiability problems (SATs), then employs SAT solvers to automatically detect security vulnerabilities within protocols; Tree Automata-based Protocol Analyzer (TA4SP) models and analyzes protocols using tree automata theory, primarily verifying protocol security under unbounded sessions or complex message structures.

**Figure 4:**
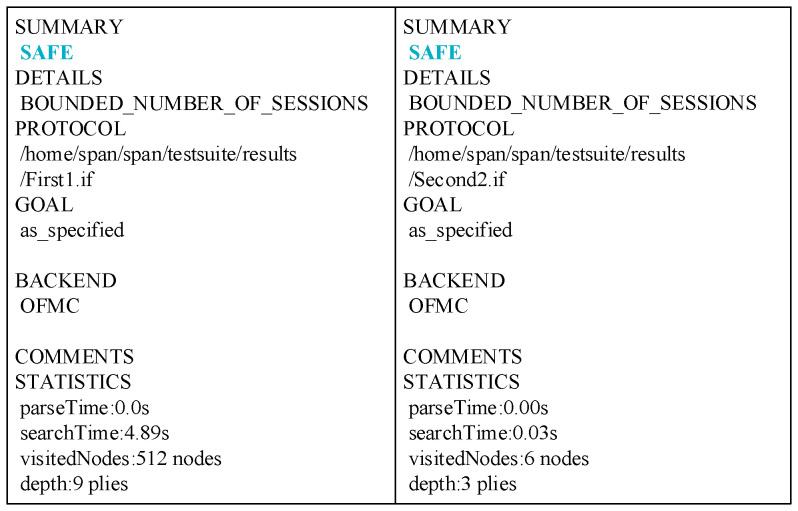
AVISPA backend module OFMC execution results.

AVISPA permits malicious adversaries to participate throughout the entire protocol interaction process during protocol analysis. These adversaries are granted the ability to intercept, tamper with, replay, and forge messages. Potential attacks they may execute include Man-in-the-Middle (MITM) attacks, type obfuscation attacks, and impersonation attacks targeting legitimate users. The simulation results of our protocol under this tool are shown in Figure 4 below. These results demonstrate that our protocol can withstand multiple malicious attacks.

### 5.4. Informal Security Analysis

In this section, we demonstrate the security features of the proposed scheme.

(1) Vehicle OBU Physical Capture Attack: Suppose adversary A reads the information $\left\{A_{i},\;B_{i},\;C_{i},\;{TID}_{V}^{i}\right\}$ stored within the OBU through a physical attack. However, the secret credentials are protected, preventing A from obtaining the registered user’s name and password, thus unable to compute the secret credentials. More importantly, we employ a PUF for defense in the information construction. The PUF generates a unique and irreplicable identifier based on the unavoidable microscopic variations inherent in the vehicle’s hardware manufacturing process. Although environmental disturbances may cause errors in the generated identifier, error correction is achievable through measures like BCH error-correcting codes and Fuzzy Extractors. Should adversary A attempt to disassemble the OBU to clone the PUF, the PUF will cease to produce valid outputs, rendering it inoperable. Consequently, adversary A cannot reconstruct the PUF.
(2) Replay Attack: We apply the timestamp property to all communication messages involved in the mutual authentication and key establishment phases (${ts}_{1},{ts}_{2},{ts}_{3},{ts}_{4}\ldots$). If A intercepts a message sent by the sender and replays it to the receiver, this replayed message will also be rejected by the receiver due to failing to satisfy the timestamp’s freshness requirement. Therefore, our scheme is resistant to replay attacks.
(3) Impersonation Attack: If adversary A attempts to intercept messages transmitted over the public channel and impersonate a legitimate vehicle, they must first forge the information {M1, M2, Q} and attempt verification with the regional RSU. Since adversary A has not been legitimately registered with the CMC, this will result in verification failure by the regional RSU.
(4) MITM Attack: Suppose A eavesdrops on and intercepts messages in the channel and attempts to reconstruct a valid message ${(Msg}_{1},{Msg}_{2},{Msg}_{3}\ldots )$. However, since A lacks knowledge of the secret credentials between entities (such as $X_{i},\;Y_{j},\;Z_{k},$ etc.), and all messages are secured through hash operations and XOR operations, it is extremely difficult for A to accomplish this. Therefore, our scheme is resistant to such attacks.
(5) Anonymity: All entities within the system are represented by temporary identifiers (${TID}_{V}^{i},\;{TID}_{R}^{j},\;{TID}_{E}^{k}$) instead of their actual names. These TIDs are dynamically updated during each authentication process (${{TID}_{V}^{i}}^{\;new},\;{{TID}_{R}^{j}}^{\;new},\;{{TID}_{E}^{k}}^{\;new}$), ensuring that adversary A remains unaware of the entities’ true identities. Consequently, the anonymity of the entities is guaranteed.
(6) Traceability: When an entity exhibits abnormal behavior and engages in malicious actions, CMC can extract the true identity of the anomalous entity using the system private key and track it. Therefore, our scheme satisfies this characteristic.
(7) Data Integrity Protection: In the proposed scheme, message integrity verification is performed for every message transmitted over the public channel, effectively preventing malicious attackers from tampering with messages. The receiver reconstructs ${Int}_{n},\;n=1,2,3\ldots$ from all received messages. If ${{Int}_{n}}^{*}\neq {Int}_{n}(n=1,2,3\ldots )$, the message is immediately discarded, and the session with the sender is terminated.
(8) Perfect Forward Secrecy: The proposed scheme establishes a temporary session key for each communication authentication, with distinct session keys generated for different session instances. Suppose an adversary A obtains messages $M_{1}=\alpha \times P$ and $M_{3}=\beta \times P$. To derive the random numbers α and β, it must solve an ECDL problem. Since it lacks the entity’s private key, calculating $M_{2}$ and $M_{4}$ remains infeasible. Furthermore, even if the system key is compromised, the adversary cannot compute SK because it lacks knowledge of $X_{i},\;Y_{j},\;Z_{k}\;$and the vehicle’s challenge–response pairs. Thus, our scheme guarantees forward secrecy.

## 6. Performance Analysis

In this section, we analyze the performance of the proposed scheme in terms of communication overhead and computational overhead, comparing it with existing schemes. Since all compared schemes involve authentication among three entities, our analysis of the proposed scheme OS1 includes the requesting vehicle, ES, and CMC; while for scheme OS2, the entities analyzed are the requesting vehicle, the auxiliary vehicle, and CMC. The primary focus here is on evaluating the protocol’s performance during mutual authentication and key establishment phases.

### 6.1. Computation Overhead

The computational overhead is primarily determined by the time required to execute cryptographic operations during the authentication process. In Table 6, we list the relevant cryptographic operations and the respective time required for their execution.

In OS1, the requesting vehicle performed a total of eight hash encryption operations, taking 0.016 ms to execute. The edge server (ES) performed a total of eleven hash encryption operations, one elliptic curve point multiplication operation, and two symmetric encryption/decryption operations, taking 0.72 ms to execute. The cloud management center (CMC) performed a total of thirteen hash encryption operations and two symmetric encryption/decryption operations, taking 0.042 ms. In OS2, the requesting vehicle (i) performed a total of eight hash encryption operations and one elliptic curve point multiplication operation, with an execution time of 0.698 ms. The auxiliary vehicle (m) performed a total of nine hash encryption operations, one elliptic curve point multiplication operation and one symmetric encryption/decryption operation, with an execution time of 0.708 ms. The cloud management center (CMC) performed a total of thirteen hash encryption operations, two elliptic curve point multiplication operations, and one symmetric encryption/decryption operation, taking 1.398 ms to complete. Similarly, the computational overhead of other schemes is calculated using the same method. Since XOR is an atomic operation, we do not consider the time spent executing XOR here.

**Figure 5:**
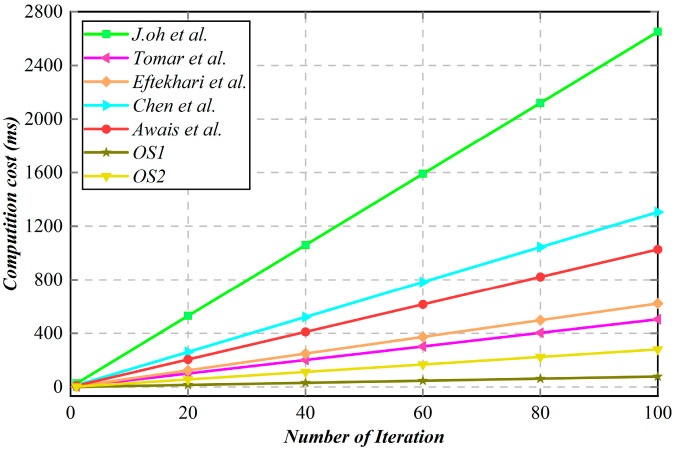
Computational overhead comparison [30,43,44,45,46].

A detailed comparison of computational overhead between the proposed scheme and other existing relevant schemes is presented in Table 7 and Figure 5. We can observe that the proposed scheme exhibits superior lightweight characteristics, aligning with the requirements of the vehicle-to-everything (V2X) computing task offloading environment.

### 6.2. Communication Overhead

Communication overhead primarily quantifies the number of message bits transmitted during a complete authentication process. We primarily investigate the communication overhead incurred during the authentication and key establishment phases. In the proposed scheme, we define the identity ID and PUF challenge response as 80 bits, the timestamp as 32 bits, and the elliptic curve point as 257 bits due to its compressibility, and message ${int}_{i}$ and $W_{i}$ are 128 bits. Additionally, the scheme employs a 256-bit hash function (SHA-256) and 256-bit random numbers.

**Figure 6:**
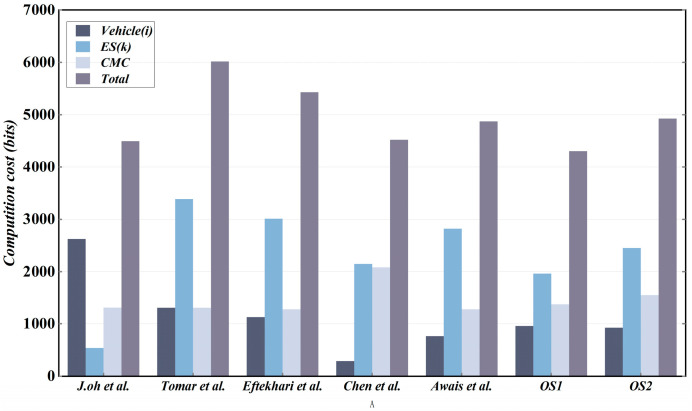
Comparative analysis of communication overhead [30,43,44,45,46].

In OS1, the messages that need to be exchanged between the requesting vehicle, ES, and CMC include ${Msg}_{1}^{V2I}=\{{Auth}_{V}^{i},\;U_{1},\;{TID}_{V}^{i},\;{Int}_{1},\;{ts}_{1}\}$, ${Msg}_{3}^{V2I}=\{E_{1},\;{TID}_{E}^{k},\;{Int}_{3},\;{ts}_{3}\}$, ${Msg}_{4}^{V2I}=\{E_{2},\;{Int}_{4},\;{ts}_{4}\}$, and ${Msg}_{7}^{V2I}=\{{U_{5}}^{*},\;U_{8},\;{Int}_{6},\;{ts}_{7}\}$. The sizes corresponding to the messages are (256 + 288 + 256+ 128 + 32 = 960 bits), (800 + 256 + 128 + 32 = 1216 bits), (1392 + 128 + 32 = 1552 bits), and (336 + 256 + 128 +32 = 752 bits), respectively; therefore, the total communication overhead is 4480 bits. Similarly, in OS2, the messages that need to be exchanged between the requesting vehicle, the auxiliary vehicle, and the CMC include ${Msg}_{1}^{V2V}=\{V_{1},\;{Auth}_{V}^{i},\;{TID}_{V}^{i},W_{1},\;{ts}_{1}\}$, ${Msg}_{2}^{V2V}=\{{W_{2},\;V}_{1},\;{Auth}_{V}^{i},\;{TID}_{V}^{i},V_{2},\;{Auth}_{V}^{m},\;{TID}_{V}^{m},\;{ts}_{2}\}$, ${Msg}_{3}^{V2V}=\{S_{1},{Int}_{3},\;{ts}_{3}\}$, and ${Msg}_{4}^{V2V}=\{{V_{5}}^{*},\;{V_{6}}^{*},W_{4},{ts}_{4}\}$. The sizes corresponding to the messages are (256 + 256+ 256+ 128 + 32 = 928 bits), (128 + 256 + 256 + 256 + 256 + 256 + 256 + 32 = 1696 bits), (1216 + 128 + 32 = 1376 bits), and (256 + 336 + 128 +32 = 752 bits), respectively; as a result, the communication overhead in OS2 is 4752 bits. For the other schemes we compare, we employ the same computational approach. A comparative analysis of communication overhead is presented in Table 8 and Figure 6.

### 6.3. Comparison of Security Features

This section compares the security features of the proposed solution with those of the existing scheme. Table 9 summarizes the security characteristics of the schemes.

## 7. Simulation Using NS-3

NS-3 [47] is a discrete-event-driven, open-source network simulator that offers outstanding functionality and flexibility. With its extensive model library and modular design, NS-3 provides powerful extensibility. In this section, we employ NS-3 to conduct simulation studies on the proposed scheme, evaluating its performance at the network layer.

### 7.1. Simulation Environment

The relevant network parameters used in the simulation process are shown in Table 10 below. The entities involved in the simulation include vehicles, ES, and CMC, with OS1 serving as an example. In the proposed scheme, the communication overhead for messages {${Msg}_{1}^{V2I}$,${Msg}_{3}^{V2I}$,${Msg}_{4}^{V2I}$,${Msg}_{7}^{V2I}$,${Msg}_{8}^{V2I}$} is {120 bytes, 152 bytes, 194 bytes, 94 bytes, 36bytes}, respectively. In scenarios 1, 2, and 3, we compared the performance resulting from varying numbers of vehicles and ESs. We evaluate the proposed scheme using three performance metrics: End-to-End Delay (EED), throughput, and Packet Delivery Rate (PDR).

### 7.2. End–End Delay (EED)

**Figure 7:**
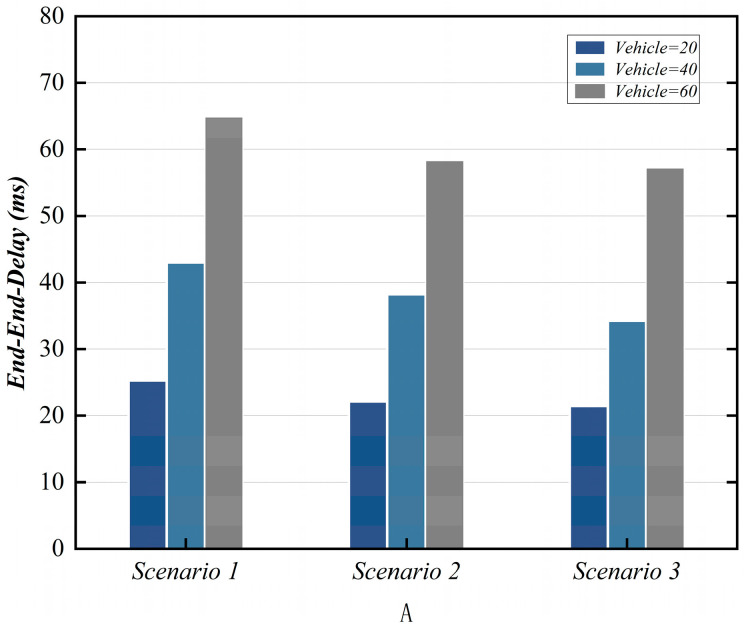
EED comparative analysis.

EED measures the average time taken for a data packet to travel from its source point (sender) to its destination point (receiver). Its calculation method is defined as $EED=\frac{\sum_{i=1}^{N_{p}}T_{{se}_{i}}-T_{{re}_{i}}}{N_{p}}$. Here, $N_{p}$ denotes the total number of packets, $T_{{se}_{i}}$ represents the transmission time of packet i, and $T_{{re}_{i}}$ denotes the reception time of packet i. Figure 7 shows the performance of EED under three scenarios.

### 7.3. Throughput

**Figure 8:**
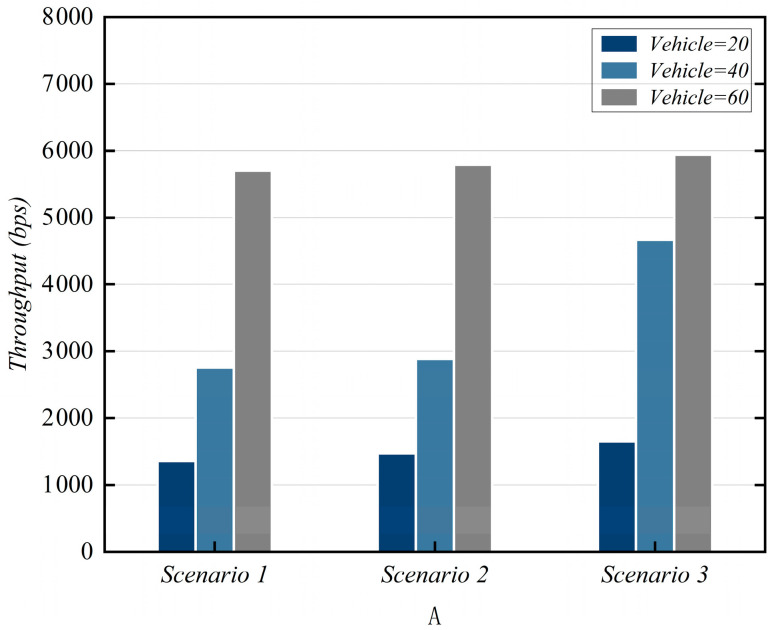
Throughput simulation results.

Throughput is defined as the amount of effective data transmitted within a network per unit of time. It serves as another core metric for measuring network performance, fundamentally differing from EED. The calculation method for throughput is defined as $Throughput=\frac{\sum_{p=1}^{N_{re}}{Size}_{p}}{T_{t}}$. Here, $N_{re}$ denotes the number of received data packets, ${Size}_{p}$ represents the size of the received data packets, and $T_{t}$ indicates the total duration of the statistical period. The throughput performance under the three scenarios is shown in Figure 8.

### 7.4. Packet Delivery Rate (PDR)

**Figure 9:**
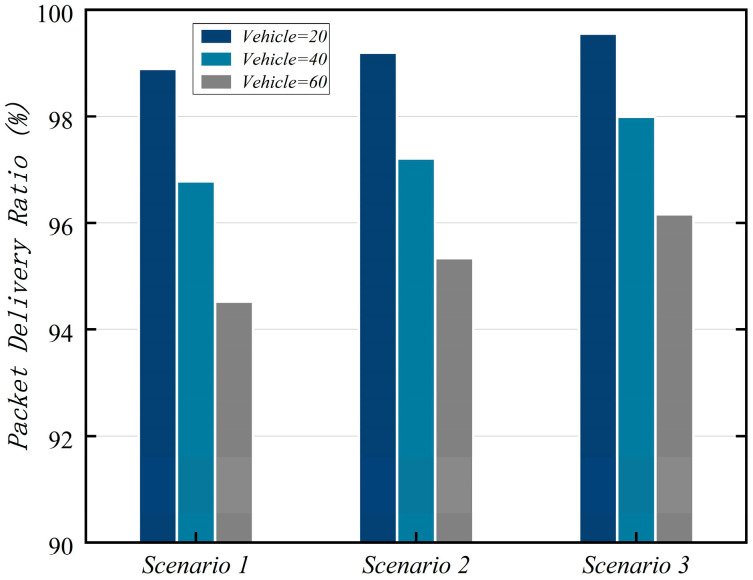
PDR simulation results.

PDR is another core metric for measuring network stability and reliability, distinct from EED and throughput. Its mathematical representation for calculation is $PDR=\frac{N_{re}^{suc}}{N_{se}}$. It is defined as the ratio of the number of packets successfully received by the destination (receiver) to the number of packets transmitted by the source (sender) within a specific time period. Figure 9 shows PDR performance under three scenarios.

## 8. Conclusions

To address security challenges in the offloading environment for vehicle-to-everything (V2X) computing tasks, we propose an authentication scheme among relevant entities involved in the offloading process. Rigorous analysis and evaluation demonstrate that this scheme satisfies the security properties required for V2X task offloading, effectively resisting common threats and attacks while safeguarding the legitimacy and privacy of the offloading process. However, significant work remains to establish a comprehensive, multi-layered security framework for vehicle-to-everything (V2X) computing task offloading.

The proposed scheme may face non-linear scalability issues under exponentially increasing vehicle traffic, potentially overloading the CMC. Therefore, future research will explore developing a dynamic mechanism based on this scheme that supports reauthentication and rapid authentication, making it more adaptable to high-speed, mobile network topologies. Second, even legitimate communication entities may exhibit malicious behavior during prolonged network interactions. Therefore, future research will focus on deeply integrating the foundational authentication proposed in this paper with higher-level behavioral trust management (e.g., PPRU and TCDEM). We aim to design a trust evaluation framework tailored for offloading computing tasks, thereby enhancing the security framework for V2X computing task offloading.

Additionally, in our research on V2X computing task offloading strategies, we will consider incorporating the computational and communication overhead generated by authentication into the strategy. We will establish a dynamic trust mechanism to achieve joint optimization of security and efficiency, thereby enhancing the reliability of V2X computing task offloading.

## Figures and Tables

**Table 1:** A comparative summary of existing authentication schemes.

Scheme	Year	Concept	Advantages	Drawbacks
Cui et al. [9]	2019	* Elliptic curve cryptography * Hash function * Elliptic curve Diffie–Hellman	Ensures anonymity Achieves traceability Perfect forward secrecy	- Multi-collaboration may add complexity - Communication delays may increase
Li et al. [5]	2020	* Hash function	Low computation overhead Support for unlinkability and anonymity	- Unrealized forward security - Idealized OBUs without considering realistic physical layer attacks
Han et al. [7]	2020	* Bilinear pairing operation * Hash function	Meet vehicle anonymity and traceability Information integrity and non-forgeability are achieved	- High computational cost
Othman et al. [22]	2021	* Elliptic curve cryptograph * Hash function and Hash-based Message Authentication Code (HAMC) * Pseudo-random function	Immune to MITM attacks Resiliency against desynchronization attacks Protection against physical attacks	- Execution of bivariate polynomials may be slow on resource-constrained OBUs
Li et al. [24]	2022	* Hash function	Satisfying forward security Resist insider attacks	- Linkability of temporary identities - Defense that does not take physical attacks into account
Wei et al. [17]	2021	* Elliptic curve cryptograph * Lagrange interpolation theorem	Multi-TA model Enhance resilience against DoS attacks Satisfying unlinkability	- Deployment cost and management complexity of sub-TA can be high
Salem et al. [28]	2023	* Elliptic curve Diffie–Hellman key exchange (ECDH) * Hash function	Supports anonymity, mutual authentication, and traceability	- No timestamp introduced - Possible offline dictionary attack
Liang et al. [29]	2023	* Elliptic curve cryptograph * Hash function * Physically unclonable function	Resistance to cloning and physical attacks Resistance to known session key attacks	- PUFs are seen as idealized, while the reality of PUFs is susceptible to environmental influences
Awais et al. [30]	2024	* Hash function * Symmetric encryption and decryption * Physically unclonable function	Resisting RSU forgery attacks Vehicle traceability Resistance to physical attacks	- Missing key confirmation step - Long-term homogeneity of the “CRP”

**Table 2:** Symbols in the scheme.

Symbols	Description
${ID}_{V}^{i},\;{ID}_{RSU}^{j},\;{ID}_{ES}^{k},\;{ID}_{CMC}$	Real identity of vehicles, RSUs, ESs, and CMC
${PK}_{sys},\;S$	The system’s public key and private key
(Cha, Res)	Challenges and responses to PUF
${fp}_{U}$	The fingerprint information
${ID}_{U},\;{PWD}_{U}$	User ID and password
$S_{V}^{i},\;S_{R}^{j},\;S_{E}^{k}$	Partial private keys of vehicles, RSUs, and ESs
${TID}_{V}^{i},\;{TID}_{R}^{j},\;{TID}_{E}^{k}$	Temporary identity of vehicles, RSUs, and ESs
${PK}_{V}^{i},\;{PK}_{R}^{j},\;{PK}_{E}^{k}$	Partial public keys of vehicles, RSUs, and ESs
Request, ACK	Request and response information
Gen()/Rep()	Cryptographic functions
${ts}_{i}$	Timestamp
⨁	XOR operation
∥	Concatenation

**Table 3:** Key concepts of BAN logic.

Concepts	Description
A|≡M	A believes M.
A| ~ M	A once said M.
A⊲M	A met M.
#(M)	M is fresh.
A|⇒M	A has jurisdiction over M.
${\langle X\rangle }_{Y},(X,Y)$	The combination of X and Y.
${\left\{M\right\}}_{K}$	The message M encrypted with key K.
$A\overset{\;K\;}{↔}B$	K is the shared key between A and B.

**Table 4:** Rules for inference in BAN logic.

Inference Rules	Form of Expression
(R1) Message-Meaning Rule	$\frac{A|\equiv A\overset{\;K\;}{↔}B,A⊲{\left\{M\right\}}_{K}}{A|\equiv B|\sim M}$
(R2) Nonce-Verification Rule	$\frac{A|\equiv \#(M),A|\equiv B|\sim M}{A|\equiv B|\equiv M}$
(R3) Jurisdiction Rule	$\frac{A|\equiv B|\Rightarrow M,A|\equiv B|\equiv M}{A|\equiv M}$
(R4) Freshness Rule	$\frac{A|\equiv \#(X)}{A|\equiv \#(X,Y)}$
(R5) Belief Rule	$\frac{A|\equiv X,A|\equiv Y}{A|\equiv (X,Y)}$

**Table 5:** Queries executable by adversary A.

Queries	Description
*Execute* ($\Pi_{V}^{i},\;\Pi_{RSU}^{j},\;\Pi_{ES}^{k},\;\Pi_{CMC}^{l}$)	This query simulates adversary A’s passive eavesdropping capability. When A invokes this query, challenger C will return messages transmitted among ($\Pi_{V}^{i},\Pi_{RSU}^{j},\Pi_{ES}^{k},\Pi_{CMC}^{l}$) over the public channel.
*Send(* $\Pi_{\Delta }^{ο},Msg$ *)*	After A sends a request message Msg to $\Pi_{\Delta }^{ο}$, C will return the corresponding response message to A.
*Corrupt(* $\Pi_{V}^{i}$ *)*	By executing this query, A can obtain the secret parameters stored in the vehicle’s OBU.
*Reveal(* $\Pi_{\Delta }^{ο}$ *)*	When A initiates a Reveal query against $\Pi_{\Delta }^{ο}$, C checks $\Pi_{\Delta }^{ο}$‘s state; if its state is accepted, C returns the session key (SK) established between $\Pi_{\Delta }^{ο}$ and its partner, otherwise, C returns null.
*Test(* $\Pi_{\Delta }^{ο}$ *)*	This query may only be invoked once by A, and $\Pi_{\Delta }^{ο}$ must satisfy the freshness requirement. When A invokes this query, C randomly selects a bit b∈{0, 1}. If b = 1, C returns the actual session key established with its partner; if b = 0, C returns to A a random string of equal length to the session key.

**Table 6:** Cryptographic operation execution time.

Operation	Description	Execute Time (ms)
$T_{H}$	The execution time of a one-way hash function.	0.002
$T_{EPA}$	The execution time of this elliptic curve addition operation.	0.004
$T_{EPM}$	The execution time of this elliptic scalar multiplication operation.	0.682
$T_{BP}$	The execution time of the bilinear pairing.	2.904
$T_{E}/T_{D}$	The execution time of this symmetric encryption and decryption.	0.008

**Table 7:** Comparative analysis of scheme computational overhead.

Scheme	Vehicles/Users	ES/Fog Node	CMC/Cloud Server
J. Oh et al. [43]	$12T_{H}+5T_{EPM}+4T_{BP}\approx 15.05\;ms$	$1T_{H}+3T_{EPM}+3T_{BP}\approx 10.76\;ms$	$10T_{H}+1T_{EPM}\approx 0.702\;ms$
Tomar et al. [44]	$8T_{H}+2T_{EPM}\approx 1.38\;ms$	$5T_{H}+2T_{EPM}\approx 1.374\;ms$	$9T_{H}+10/3T_{EPM}\approx 2.291\;ms$
Eftekhari et al. [45]	$11T_{H}+3T_{EPM}+1T_{EPA}\approx 2.072\;ms$	$12T_{H}+3T_{EPM}+1T_{EPA}\approx 2.074\;ms$	$15T_{H}+3T_{EPM}+2T_{EPA}\approx 2.084\;ms$
Chen et al. [46]	$9T_{H}+4T_{EPM}+1T_{E}/T_{D}\approx 2.754\;ms$	$8T_{H}+7T_{EPM}+3T_{EPA}+1T_{E}/T_{D}\approx 4.81\;ms$	$9T_{H}+8T_{EPM}+2T_{EPA}+1T_{E}/T_{D}\approx 5.49\;ms$
Awais et al. [30]	$7T_{H}+4T_{EPM}\approx 2.742\;ms$	$4T_{H}+5T_{EPM}\approx 3.418\;ms$	$9T_{H}+6T_{EPM}\approx 4.11\;ms$
OS1	${8T}_{H}\approx 0.016\;ms$	*11* $T_{H}+1T_{EPM}+2T_{E}/T_{D}\approx 0.72\;ms$	$13T_{H}+2T_{E}/T_{D}\approx 0.042\;ms$
OS2	$8T_{H}+1T_{EPM}\approx 0.698\;ms$	$9T_{H}+1T_{EPM}+1T_{E}/T_{D}\approx 0.708\;ms$	*13* $T_{H}+2T_{EPM}+1T_{E}/T_{D}\approx 1.398\;ms$

**Table 8:** Communication overhead of participating entities.

Scheme	Vehicles/Users	ES/Fog Node	CMC/Cloud Server
J. Oh et al. [43]	2629 bits	546 bits	1316 bits
Tomar et al. [44]	1312 bits	3392 bits	1312 bits
Eftekhari et al. [45]	1137 bits	3012 bits	1281 bits
Chen et al. [46]	288 bits	2148 bits	2084 bits
Awais et al. [30]	769 bits	2822 bits	1283 bits
OS1	960 bits	1968 bits	1376 bits
OS2	928 bits	2448 bits	1552 bits

**Table 9:** Security features analysis.

Security Attributes	J. Oh et al. [43]	Tomar et al. [44]	Eftekhari et al. [45]	Chen et al. [46]	Awais et al. [30]	OS1/OS2
SA-1	YES	YES	YES	NO	YES	YES
SA-2	NO	NO	NO	NO	NO	YES
SA-3	YES	YES	YES	NO	NO	YES
SA-4	YES	YES	YES	YES	YES	YES
SA-5	YES	YES	YES	YES	YES	YES
SA-6	YES	NO	YES	YES	YES	YES
SA-7	YES	YES	YES	YES	YES	YES
SA-8	NO	NO	NO	YES	NO	YES
SA-9	YES	YES	YES	YES	YES	YES

Note: SA-1: “Entity Anonymity”; SA-2: “Capture Physical Attack”; SA-3: “Reply Attack”; SA-4: “Mutual Authentication”; SA-5: “MITM Attack”; SA-6: “Entity Impersonation Attack”; SA-7: “Traceability”; SA-8: “Integrity Verify”; SA-9: “Privileged Insider Attack”. (“YES” indicates possession; “NO” indicates non-possession.)

**Table 10:** Simulation parameters.

Parameters	Value
Platform	Ubuntu 20.04
Simulation tool	NS-3 (3.33)
Routing protocol	OLSR
Network area	2 km
Number of vehicles	(20, 40, 60)
Number of ESs	5 (Scenario 1), 7 (Scenario 2), 9 (Scenario 3)
Number of CMC	1 (Scenario 1, Scenario 2, Scenario 3)
Mobility of vehicles	(0–54 km/h)
Protocol standard	IEEE 802.11 [48]
Simulation time	800 s

## Data Availability

Data are contained within the article.
